# The Stricter the Better? The Relationship between Targeted HbA_1c_ Values and Metabolic Control of Pediatric Type 1 Diabetes Mellitus

**DOI:** 10.1155/2016/5490258

**Published:** 2016-01-05

**Authors:** Marcin Braun, Bartlomiej Tomasik, Ewa Wrona, Wojciech Fendler, Przemyslawa Jarosz-Chobot, Agnieszka Szadkowska, Agnieszka Zmysłowska, Jayne Wilson, Wojciech Mlynarski

**Affiliations:** ^1^Department of Pediatrics, Oncology, Hematology and Diabetology, Medical University of Lodz, Sporna 36/50, 91-738 Lodz, Poland; ^2^Department of Pediatrics, Endocrinology and Diabetology, Medical University of Silesia, Medykow 16, 40-752 Katowice, Poland; ^3^Cancer Research UK Clinical Trials Unit, School of Cancer Sciences, University of Birmingham, Vincent Drive, Edgbaston, Birmingham B15 2TT, UK

## Abstract

*Introduction*. It remains unclear how HbA_1c_ recommendations influence metabolic control of paediatric patients with type 1 diabetes mellitus. To evaluate this we compared reported HbA_1c_ with guideline thresholds.* Materials and Methods*. We searched systematically MEDLINE and EMBASE for studies reporting on HbA_1c_ in children with T1DM and grouped them according to targeted HbA_1c_ obtained from regional guidelines. We assessed the discrepancies in the metabolic control between these groups by comparing mean HbA_1c_ extracted from each study and the differences between actual and targeted HbA_1c_.* Results*. We included 105 from 1365 searched studies. The median (IQR) HbA_1c_ for the study population was 8.30% (8.00%–8.70%) and was lower in “6.5%” than in “7.5%” as targeted HbA_1c_ level (8.20% (7.85%–8.57%) versus 8.40% (8.20%–8.80%); *p* = 0.028). Median difference between actual and targeted HbA_1c_ was 1.20% (0.80%–1.70%) and was higher in “6.5%” than in “7.5%” (1.70% (1.30%–2.07%) versus 0.90% (0.70%–1.30%), resp.; *p* < 0.001).* Conclusions*. Our study indicates that the 7.5% threshold results in HbA_1c_ levels being closer to the therapeutic goal, but the actual values are still higher than those observed in the “6.5%” group. A meta-analysis of raw data from national registries or a prospective study comparing both approaches is warranted as the next step to examine this subject further.

## 1. Introduction

Despite the crucial role of HbA_1c_ in the management of diabetes, substantial differences among diabetic associations regarding targeted levels of this parameter are still present. The stricter approach is represented by the European Society of Cardiology (ESC) and recommends 6.5% (48 mmol/mol) of HbA_1c_ [1], whilst the International Society for Pediatric and Adolescent Diabetes (ISPAD), International Diabetes Federation (IDF), and American Diabetes Association (ADA) advocate 7.5% (58 mmol/mol) as the valid target of HbA_1c_ [2, 3].

The Hvidøre Study Group on Childhood Diabetes observed significant differences in average HbA_1c_ levels among 21 large pediatric diabetes centres from 17 countries in Europe, Japan, and North America [4]. The authors of this study suggested that there might be several reasons for these discrepancies. One possible explanation might be bound to guideline values of desirable HbA_1c_ level that differ depending on the diabetic association.

Both the Diabetes Control and Complications Trial (DCCT) and the Epidemiology of Diabetes Interventions and Complications Study (EDIC) have shown that early intensive therapy of patients with type 1 diabetes results in better metabolic control [5, 6]. The differences in metabolic control between conventional therapy and functional intensive insulin treatment cohorts were directly correlated with strikingly different treatment outcomes (e.g., any cardiovascular disease event was reduced by 42% in the intensive metabolic control arm). Taking into consideration the results of DCCT and EDIC studies, if the difference among targeted HbA_1c_ levels of 1% was related to a different metabolic control, this would have an impact on long-term complications of diabetes mellitus. Recent cohort studies have shown that metabolic control of patients with T1DM has significantly improved during the last decade and better metabolic control resulted in superior outcome which supports aforementioned observations [7, 8].

In view of the controversy of the role of HbA_1c_ guidelines and the fact that, in majority of cases, they are set arbitrarily, we have conducted this study to examine the influence of HbA_1c_ targets on metabolic control of type 1 diabetes in pediatric population [9].

## 2. Materials and Methods

### 2.1. Guideline Identification

The data on guideline HbA_1c_ values in each country or region at the time of the study were obtained from official websites of national/regional diabetic associations. In case of lack of information consultants were contacted by phone or e-mail.

### 2.2. Reported HbA_1c_ Levels: Data Sources and Searches

Publications which reported HbA_1c_ levels were sought using systematic review techniques. A systematic search was undertaken using terms for pediatric/children/juvenile diabetes mellitus type 1, glycated haemoglobin A_1c_, and insulin-based therapy in the following databases: OVID MEDLINE, EMBASE, Cochrane Database of Systematic Reviews (CDSR), National Institute for Health and Clinical Excellence (NICE) database, Scottish Intercollegiate Guidelines (SIGN) database, Database of Reviews of Effects (DARE), and Health Technology Assessment (HTA/NHS EED). The searches were conducted between 1st January 2008 and 26th August 2013, with no language restrictions. We searched for studies with T1DM pediatric patients (≤18 years) being treated for at least 1 year. The patients had to have at least one HbA_1c_ level measurement taken after December 2007. In cases of interventional studies, we utilized the HbA_1c_ levels recorded prior to the planned intervention. We excluded studies with less than 50 participants. The study design and search strategy are available in the Supporting Information, available online at http://dx.doi.org/10.1155/2016/5490258.

### 2.3. Study Selection

Two reviewers independently assessed searched papers for eligibility: firstly by screening titles and abstracts and secondly by examining full-text papers of studies included after screening. The same strategy was used for both data bits' extraction. All disagreements were resolved by the discussion between reviewers.

In order to reduce bias caused by overlapping studies we contacted corresponding authors of papers, which we found to be based on common national diabetic registries or which had at least one common author or when the research was done in the same institution. When we detected that the results of several papers overlapped each other and we could not obtain separated data, we chose the study with the largest sample.

### 2.4. Data Extraction

Data were extracted onto a predefined form. HbA_1c_ levels representing populations of each study were calculated by combining the mean values for all investigated groups. If applicable, all values were transformed and presented as percentage of the total haemoglobin level by using standard HbA_1c_ units' converter [10]. HbA_1c_ measurements had to be performed using high-performance liquid chromatography meeting the DCCT standard. For interventional studies HbA_1c_ values were extracted before implementation of preplanned intervention.

### 2.5. Data Synthesis and Statistical Analysis

Subgroup analyses were undertaken according to the following: gross domestic product (GDP; high-income country is defined to have a GDP above US$12,746 in 2013) [11], number of patients in each study, age, duration of type 1 diabetes mellitus, prevalence of acute complications (hypoglycaemia and ketoacidosis), and type of therapy (continuous subcutaneous insulin infusion and multiple daily injection). If studies were conducted across multiple countries we checked whether guideline values were homogenous among them and allocated to appropriate groups. If targeted values were different in individual studies and we could not extract separate data for each country we excluded such studies from the quantitative analyses. In order to compare compliance, the difference between actual and targeted guideline HbA_1c_ value was calculated for each study (ΔHbA_1c_).

Nominal variables were given as numbers with appropriate percentage whereas continuous variables were given as medians with interquartile ranges (IQR). For pairwise comparisons of continuous variables, the Mann-Whitney U (MWU) test was used. For multigroup comparisons Kruskal-Wallis one-way analysis of variance with additional post hoc tests within subgroups was used. Correlations were assessed using Spearman's rank correlation coefficient. Multivariate analyses were made using linear regression (GLM) weighted by the logarithm of number of participants in included studies. The multivariate model was fitted including all the variables that had reached statistical significance in the univariate analyses. Meta-analysis for comparison of adherence to guideline values was conducted using differences between mean and guideline HbA_1c_ levels. *I* ^2^ values to assess the heterogeneity between studies were calculated and results above 50% were considered as being of high heterogeneity. Statistical significance was set at *p* ≤ 0.05; 95% confidence intervals were calculated. Statistical analysis was performed with usage of STATISTICA 10.0 software (StatSoft, Tulsa, OK, USA).

## 3. Results

### 3.1. Studies' Selection

**Figure 1:**
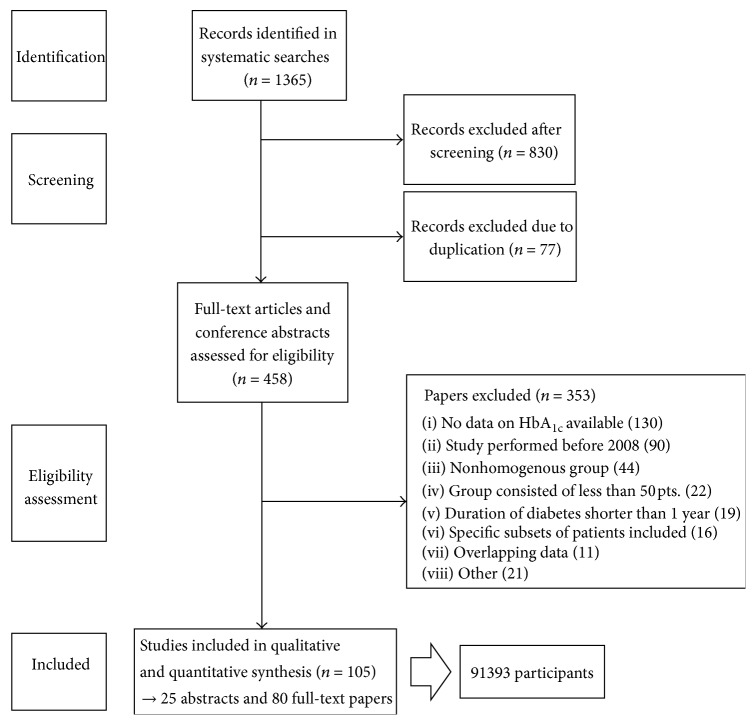
Flowchart for studies' selection process.

The systematic searches for published HbA_1c_ levels yielded 1365 records. Eight hundred thirty of them were excluded after screening by title and abstract and further 77 were excluded due to duplication. Three hundred and fifty-three papers were excluded after full-text paper analysis. One hundred five studies were included for the final analyses, involving 91393 patients. In 25 studies only the abstract was available. The flowchart for the study selection process with detailed reasons for exclusion is presented in Figure 1.

### 3.2. Studies' Characteristics and Allocation of Countries and Regions regarding Guideline Values

Of the 105 studies yielding HbA_1c_ level data 47 (44.76%) were cross-sectional, 40 (38.10%) were cohort, 9 (8.57%) were case-series or case-control, and 9 (8.57%) were interventional (including 5 randomized clinical trials). The eligible studies came from 18 countries (at least one country from Africa, Asia, Australia, Europe, South America, and North America). Forty-three (40.95%) studies were from European Union (21 of them (48.84%) were represented by 3 countries—UK (9 studies), Poland (6 studies), and Germany (6 studies)), whilst 39 (37.14%) were from the USA. Fifty-five (52.38%) studies were allocated to the group of “7.5% (58 mmol/mol)” as guideline HbA_1c_ value; 42 (40.00%) were allocated to the group of “6.5% (48 mmol/mol).” The guideline values from remaining studies (7.62%) were not homogenous and thus we excluded them from further analyses (the complete data for comparisons among all three study groups are available in Supporting Information). Ninety-five (90.48%) studies were conducted in high-income countries. The smallest study enrolled 50 patients [12], whilst the biggest one included 42881 individuals (cross-sectional study from Germany, Austria, and Switzerland) [13]. The median number of enrolled patients was 146 (IQR: 90–368). Median age within the studies was 12.79 (IQR: 11.60–13.77) years and median duration of diabetes was 5.20 (IQR: 3.90–6.30) years. In 26 studies (24.76%) patients were treated more frequently with MDI; in 30 (28.57%) of studies CSII was the preferred method of therapy. Only in 5 (4.76%) and in 8 (7.62%) of included studies did acute complications of diabetes mellitus (hypoglycaemia and diabetic ketoacidosis, resp.) occur more frequently in comparison to the prevalence reported in the literature. A detailed table with studies' characteristics and references can be seen in Supporting Information.

### 3.3. Comparison of HbA_1c_ Levels regarding HbA_1c_ Guideline Values

**Figure 2:**
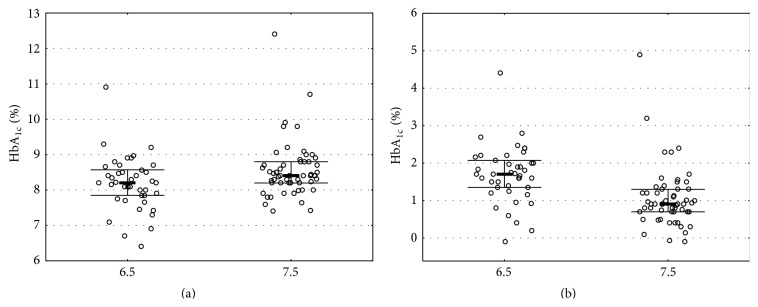
(a) Comparison for HbA_1c_ levels between regions of 6.5% and 7.5% as guideline values (MWU test, *p* = 0.0162). (b) Comparison of the difference between HbA_1c_ levels and guideline HbA_1c_ values between regions of 6.5% and 7.5% as guideline values (MWU test, *p* < 0.0001). Bolded line represents median; whiskers represent IQR.

The median (IQR) HbA_1c_ level in the whole study population was 8.30% (IQR: 8.00%–8.70%) (67 (IQR: 63–72) mmol/mol). Median values for HbA_1c_ in groups regarding guideline values were significantly lower in “6.5” group than in “7.5” group and equalled 8.20% (IQR: 7.85%–8.57%; 66 (IQR: 62–70) mmol/mol) versus 8.40% (IQR: 8.20%–8.80%) (68 (IQR: 66–73) mmol/mol), respectively (MWU—*p* = 0.028, Figure 2(a); GLM—*p* = 0.001; beta for “6.5” group = −0.22 (95% CI: −0.34, −0.09)). This difference was significant in linear regression model with studies weighted by logarithm from the number of patients (*p* = 0.025, beta for “6.5” group = −0.16 (95% CI: −0.29, −0.22)).

### 3.4. Comparison of the Difference between HbA_1c_ Levels and Guideline HbA_1c_ Values

The median ΔHbA_1c_ in the whole study population was 1.20% (IQR: 0.80%–1.70%). Median values for ΔHbA_1c_ in groups regarding guideline values were significantly higher in “6.5” group than in “7.5” group and equalled 1.70% (IQR: 1.30%–2.07%) versus 0.9% (IQR: 0.70%–1.30%) respectively (MWU—*p* < 0.001, Figure 2(b); GLM—*p* < 0.001; beta for “6.5” group = 0.40). The forest plots for meta-analysis of ΔHbA_1c_ are available in the Supporting Information.

### 3.5. Evaluation of the Effect of Other Variables on HbA_1c_ Level

Study design, publication type, percentage of patients with severe diabetic ketoacidosis or severe hypoglycaemia, type of therapy, GDP per capita, and number of patients did not have a significant impact on HbA_1c_ levels. HbA_1c_ values were significantly lower in countries from Europe (in comparison with the USA). We observed a positive correlation between HbA_1c_ levels and duration of type 1 diabetes and age of patients. All results for discussed comparisons are available in Table 1.

## 4. Discussion

Taking into consideration the debate regarding the role of HbA_1c_ guidelines and the fact that in majority of cases they are set arbitrarily, we decided to conduct this study to examine the impact of HbA_1c_ targets on metabolic control of type 1 diabetes in pediatric population [14]. Our work suggests that patients treated in centres with lower HbA_1c_ targets have better metabolic control despite being further from reaching their goal than patients from higher target countries. We found that the diabetic populations in countries with 6.5% (48 mmol/mol) as the targeted HbA_1c_ values are represented by actual median levels of HbA_1c_ of 0.2% lower than countries with 7.5% (58 mmol/mol) as targeted levels. Although the adherence was better in the centres with less strict aims of the therapy (median ΔHbA_1c_ 1.70% (1.30%–2.07%) versus 0.90% (0.70%–1.30%), resp.) the final outcome in terms of metabolic control was better in the more strict centres. We found also discrepancies between centres included in our study and this result tends to agree with the main observation of the Hvidøre Study Group [4].

The real reason for discrepancies between guideline levels for HbA_1c_ is likely to be linked to different aims and priorities in the management of diabetes [4, 15]. The teams, which set higher HbA_1c_ goals, are more concerned about risks of intensive therapy, such as hypoglycaemia. Although the previously strong association of low HbA_1c_ with severe hypoglycemia in young individuals with type 1 diabetes has substantially decreased in the last decade [16] this complication is one of the most common fatal acute diabetic complications [17]. Therefore the supporters of less strict metabolic control are willing to make concessions in order to avoid the risk. Higher HbA_1c_ values are also easier to accept and may lead to a better compliance. On the other hand, other teams focus on avoidance of hyperglycaemia, because they are devoted to minimalize the risk of long-term complications from the very first day after the diagnosis of diabetes mellitus. The crucial issue of this approach is to convince patients and their families to put an effort in pursuing stricter metabolic control [15, 18]. Additionally, since recently developed technologies including insulin pumps with low glucose suspend software reduce the risk of hypoglycaemia, the restricted goal is safely achievable [19, 20].

The differences in average HbA_1c_ levels among large diabetic centres have been the area of interest of researchers in pediatric diabetology for years [4, 15, 21–23]. The authors from the Hvidøre Study Group on Childhood Diabetes indicated several reasons, which could lead to discrepancies. The majority of these issues are relatively hard to assess in a robust way (e.g., the role of multidisciplinary approach, self-care behaviours, educational models, ethnic or cultural aspects, socioeconomic status, etc.) [24]. Recent findings suggest that the phenomenon of seasonal HbA_1c_ variability in schoolchildren could also affect the results of reported HbA_1c_ results [25]. According to Hvidøre Study Group different guideline values of HbA_1c_ among centres also appear to play a significant role in explaining the differences in metabolic outcomes among pediatric population [15]. The results of our study supports this hypothesis.

Although this is the first study that systematically examines the influence of different guideline HbA_1c_ values on the actual HbA_1c_ levels, it should be stated that our work has several limitations. The problem of overlapping populations in the enrolled studies was particularly hard to eliminate in our review. Although we tried to contact with the authors of the studies, in which such problem might have occurred, the response rate level was lower than 20%. Another issue which affects our study is the fact that more than a half of enrolled studies concerned several countries. Studies from United States of America constituted 37% of all included studies and studies from Poland, Germany, and United Kingdom constituted further 20% when considered together. Additionally it should be mentioned that we included both studies based on national registries (e.g., Germany and Austria or Sweden) and single centre studies. In the major analyses, which we are presenting in this report, we excluded 8 studies due to heterogenous HbA_1c_ guideline values. In our opinion such small number of studies is not representative and analysis of their results would be burdened with high risk of bias. Because we included studies of various designs, from which neither aimed directly to compare groups of different HbA_1c_ guidelines, we found linear regression model weighted by number of participants in each study as most appropriate for our comparisons. We present the results for all comparisons in the Supporting Information. Furthermore, our search was not restricted to studies that contained data on other variables that might have an impact on DMI control. Hence, the results on, for example, acute complications (ketoacidosis and hypoglycemia) were not fully covered in the included papers and our subgroup analysis should be treated with caution. Nevertheless we conducted the analyses on those aspects within representative studies. Only in 5 (4.76%) and in 8 (7.62%) of included studies hypoglycaemia and diabetic ketoacidosis occurred more frequently in comparison to the prevalence reported in the literature. Although this is an ecological study, with its inherent limitations (i.e., cause and effect cannot be proven and confounding factors cannot be eliminated) we attempted to strengthen the data used by employing systematic review techniques to identify and process the published data set that was used for HbA_1c_ levels. By using systematic review techniques for our searches we have attempted to reduce selection and publication biases and by using double data extraction we have reduced possible errors in data collection and analysis. Therefore our study is a robust piece of work for ascertaining the impact of discrepancies between major diabetic associations regarding targeted HbA_1c_ levels in children with type 1 diabetes.

## 5. Conclusions

Our study shows that the targeted HbA_1c_ level plays an important role in terms of metabolic control in children with type 1 diabetes. The consequences of this observation should be discussed among health care professionals. Target values for HbA_1c_ levels for children and adolescents suffering from type 1 diabetes vary between countries and centres which, in the light of our study, affects metabolic control of the patients and may have an impact on long-term complications in the future. Our study provides a solid basis for rational discussion on the impact of guideline HbA_1c_ values on the metabolic control. According to our findings the “6.5% approach” results in better outcomes, but other factors such as multidisciplinary approach, self-care behaviours, educational models, ethnic or cultural aspects, socioeconomic status, and seasonal variability should be taken into consideration. In our opinion a metaregression of raw data from national registries is warranted as a next step to corroborate our results and finally ascertain the effect of HbA_1c_ guideline values on the metabolic control among children with type 1 diabetes mellitus.

## Supplementary Material

We divided Supplementary materials into five sections. The first two sections demonstrate the study design and search strategy. The third part presents the results for the comparisons of HbA_1c_ and ∆HbA_1c_ within pre-planned subgroups. The fourth part displays the results from the meta-analysis of ∆HbA_1c_ with respect to HbA_1c_ guideline values. Tabularized features of the studies included in the review along with references are included in the fifth part.

## Figures and Tables

**Table 1:** Univariate and multivariate linear model results for HbA_1c_ values regarding HbA_1c_ guideline groups (6.5% and 7.5%) and the other covariates. NA means not applied.

Variable	Groups	Number of studies included in analysis	*p* value from univariate analysis	*p* value from multivariate analysis with *β* parameter (95% CI)	The effect on HbA_1c_ level
HbA_1c_ targeted levels	6.5 (48 mmol/mol) versus 7.5 (58 mmol/mol)	97	0.028	<0.001 *β* = −0.26 (−0.40, −0.12)	Lower in “6.5% (48 mmol/mol) countries”

Publication type	Article versus abstract	97	0.967	NA	NA

Hypoglycemia	More versus less frequent	30	0.427	NA	NA

Diabetic ketoacidosis	More versus less frequent	26	0.261	NA	NA

Type of therapy	MDI versus CSII	56	0.249	NA	NA

Study design	Five groups	97	0.356	NA	NA

Location	Europe versus USA	82	0.022	NA	Lower in Europe

GDP per capita ($)	Continuous variable	97	0.127	NA	NA

Number of patients in the study	Continuous variable	97	0.985	NA	NA

Mean age in the study (years)	Continuous variable	93	0.016	0.001 *β* = 0.29 (0.12, 0.47)	Positive correlation (*r* = 0.25)

Mean duration of DM in the study (years)	Continuous variable	85	0.037	0.753 *β* = −0.03 (−0.20, −0.15)	Positive correlation (*r* = 0.23)

## References

[1] http://www.escardio.org/Guidelines-&-Education/Clinical-Practice-Guidelines/Diabetes-Pre-Diabetes-and-Cardiovascular-Diseases-developed-with-the-EASD.

[2] http://www.idf.org/sites/default/files/Diabetes-in-Childhood-and-Adolescence-Guidelines.pdf.

[3] American Diabetes Association (2015). Standards of medical care in diabetes: children and adolescents. *Diabetes Care*.

[4] Danne T., Mortensen H. B., Hougaard P. (2001). Persistent differences among centers over 3 years in glycemic control and hypoglycemia in a study of 3,805 children and adolescents with type 1 diabetes from the Hvidøre Study Group. *Diabetes Care*.

[5] The Diabetes Control and Complications Trial Research Group (1993). The effect of intensive treatment of diabetes on the development and progression of long-term complications in insulin-dependent diabetes mellitus. The Diabetes Control and Complications Trial Research Group. *The New England Journal of Medicine*.

[6] Nathan D. M., Cleary P. A., Backlund J.-Y. C. (2005). Intensive diabetes treatment and cardiovascular disease in patients with type 1 diabetes. *The New England Journal of Medicine*.

[7] Dovc K., Telic S. S., Lusa L. (2014). Improved metabolic control in pediatric patients with type 1 diabetes: a nationwide prospective 12-year time trends analysis. *Diabetes Technology and Therapeutics*.

[8] Maahs D. M., Hermann J. M., DuBose S. N. (2014). Contrasting the clinical care and outcomes of 2,622 children with type 1 diabetes less than 6 years of age in the United States T1D Exchange and German/Austrian DPV registries. *Diabetologia*.

[9] Stroup D. F., Berlin J. A., Morton S. C. (2000). Meta-analysis of observational studies in epidemiology: a proposal for reporting. Meta-analysis of Observational Studies in Epidemiology (MOOSE) group. *Journal of the American Medical Association*.

[10] NSGP http://www.ngsp.org/docs/IFCCstd.pdf.

[11] http://data.worldbank.org/about/country-and-lending-groups#High_income.

[12] Juvenile Diabetes Research Foundation Continuous Glucose Monitoring Study Group (2010). Effectiveness of continuous glucose monitoring in a clinical care environment: evidence from the Juvenile Diabetes Research Foundation Continuous Glucose Monitoring (JDRF-CGM) trial. *Diabetes Care*.

[13] Rohrer T. R., Hennes P., Thon A. (2010). Down's syndrome in diabetic patients aged <20 years: an analysis of metabolic status, glycaemic control and autoimmunity in comparison with type 1 diabetes. *Diabetologia*.

[14] Jarosz-Chobot P., Polańska J., Myśliwiec M. (2012). Multicenter cross-sectional analysis of values of glycated haemoglobin (HbA1c) in Polish children and adolescents with long-term type 1 diabetes in Poland: PolPeDiab study group. *Pediatric Endocrinology, Diabetes, and Metabolism*.

[15] Swift P. G. F., Skinner T. C., de Beaufort C. E. (2010). Target setting in intensive insulin management is associated with metabolic control: the Hvidoere Childhood Diabetes Study Group Centre Differences Study 2005. *Pediatric Diabetes*.

[16] Karges B., Rosenbauer J., Kapellen T. (2014). Hemoglobin A1c levels and risk of severe hypoglycemia in children and young adults with type 1 diabetes from Germany and Austria: a trend analysis in a cohort of 37,539 patients between 1995 and 2012. *PLoS Medicine*.

[17] Morimoto A., Onda Y., Nishimura R., Sano H., Utsunomiya K., Tajima N. (2013). Cause-specific mortality trends in a nationwide population-based cohort of childhood-onset type 1 diabetes in Japan during 35 years of follow-up: the DERI Mortality study. *Diabetologia*.

[18] Boot M., Volkening L. K., Butler D. A., Laffel L. M. B. (2013). The impact of blood glucose and HbA_1c_ goals on glycaemic control in children and adolescents with Type 1 diabetes. *Diabetic Medicine*.

[19] Ly T. T., Brnabic A. J. M., Eggleston A. (2014). A cost-effectiveness analysis of sensor-augmented insulin pump therapy and automated insulin suspension versus standard pump therapy for hypoglycemic unaware patients with type 1 diabetes. *Value in Health*.

[20] Stenerson M., Cameron F., Wilson D. M. (2014). The impact of accelerometer and heart rate data on hypoglycemia mitigation in type 1 diabetes. *Journal of Diabetes Science and Technology*.

[21] Mortensen H. B., Hougaard P. (1997). Comparison of metabolic control in a cross-sectional study of 2,873 children and adolescents with IDDM from 18 countries. *Diabetes Care*.

[22] Schwartz L., Drotar D. (2006). Defining the nature and impact of goals in children and adolescents with a chronic health condition: a review of research and a theoretical framework. *Journal of Clinical Psychology in Medical Settings*.

[23] Wolpert H. A., Anderson B. J. (2001). Metabolic control matters: why is the message lost in the translation? The need for realistic goal-setting in diabetes care. *Diabetes Care*.

[24] Cameron F. J., Skinner T. C., de Beaufort C. E. (2008). Are family factors universally related to metabolic outcomes in adolescents with type 1 diabetes?. *Diabetic Medicine*.

[25] Mianowska B., Fendler W., Szadkowska A. (2011). HbA1c levels in schoolchildren with type 1 diabetes are seasonally variable and dependent on weather conditions. *Diabetologia*.

