# Supplementary material for: The Stricter the Better? The Relationship between Targeted HbA1c Values and Metabolic Control of Pediatric Type 1 Diabetes Mellitus
Source: J Diabetes Res. 2016 Jan 5;2016:5490258. doi: 10.1155/2016/5490258 (PMC4736392; doi:10.1155/2016/5490258)
Supplement: Supplementary file 1 — We divided Supplementary materials into five sections. The first two sections demonstrate the study design and search strategy. The third part presents the results for the comparisons of HbA1c and ∆HbA1c within pre-planned subgroups. The fourth part displays the results from the meta-analysis of ∆HbA1c with respect to HbA1c guideline values. Tabularized features of the studies included in the review along with references are included in the fifth part. [file 5490258.f1.pdf]

Supplementary material 1. Study protocol.

INTRODUCTION
**Nature and context of the problem:**
There are large discrepancies between diabetic associations worldwide in defining targeted value of HbA_{1c}. Taking into consideration the aforementioned fact and large differences in metabolic control of paediatric patients with type 1 diabetes mellitus, a question about the real impact of those guideline values on metabolic control still needs to be answered.
**Aim of review:**
To compare achieved HbA_{1c} concentrations with targeted guideline values in paediatric patients with DM1 worldwide.
**Rationale:**
Measurement of HbA_{1c} concentration is a valid clinical test for metabolic control of patients with diabetes mellitus. The guideline values for HbA_{1c} differ among countries, especially when paediatric population is taken into consideration. There is hardly evidence that could assess which guidelines are the most appropriate to accurately control this disease among patients under 18 yrs.. Therefore we aim in this review to compare the HbA_{1c} values after 1 yr. after diagnosis and appropriate treatment with relation to guideline values between different countries worldwide.
RESEARCH QUESTION
**Population**
Paediatric population (<18 yrs.) with diagnosed T1DM and treated with insulin (Multiple Daily Injections, Continuous Subcutaneous Insulin Infusion) for more than 1 yr.
**Reference standard**
Guideline values that were applied at the moment when the study was on-going.
**Outcomes**
HbA_{1c} concentration (mean±SD) among participants at the beginning of each study; difference (delta (Δ)) of [HbA_{1c}] values between guideline and actual HbA_{1c} value in each study; subgroups in studies with HbA_{1c} values below 10%; regarding gross-domestic product (GDP) and prevalence of acute diabetic complications
**Study designs included in our review**
Registries, interventional trials, cross-sectional trials will be included in the review as well as case series with start date in 2008 and number of participants more than 50, because such case series with lower number of patients often include preferred groups, e.g. only good metabolic control patients
SEARCH PLAN
**Scoping searches**

Scoping searches*, to identify systematic reviews and health technology assessments on this topic will be undertaken in the following:
Cochrane Database of Systematic Reviews (CDSR)
http://www.library.nhs.uk/default.aspx
Database of Reviews of Effects (DARE)
http://www.crd.york.ac.uk/crdweb/
Health Technology Assessment Database (HTA)
http://www.crd.york.ac.uk/crdweb/
Agency for Health Technology Assessment in Poland (AHTAPol) http://www.aotm.gov.pl
*based on the ARIF protocol - http://www.arif.bham.ac.uk/strategy.shtml [accessed 7-2-11]

Main review searches

The main aim of the search will be to systematically identify studies. The following data sources will be searched:

- Bibliographic databases including Cochrane Library (CENTRAL), MEDLINE, EMBASE
- Citation lists of relevant studies
- Contact with experts in the field
- Conference proceedings – any specific paediatric conferences Treatment algorithms; Guidelines
- Previous trials unit protocols.

Up to the moment guideline values will be obtained from official websites of national associations for diabetes in each of selected countries.

No language restrictions will be applied. We will take into consideration studies no older than five years. If we find a systematic review and it is reliable one (after critical appraisal) then we will narrow our search date to update the evidence we have.

Example of search strategy

Database: Embase <1996 to 2013 Week 34>
Search Strategy:
-- 1
paediatric.mp. or pediatrics/ (72936)
2 limit 1 to yr="2008 -Current" (39429)
3 pediatric.mp. or pediatrics/ (210372)
4 limit 3 to yr="2008 -Current" (116292)
5 2 or 4 (135620)
6 diabetes.mp. or diabetes mellitus/ (463736)
7 limit 6 to yr="2008 -Current" (259967)
8 insulin.mp. or insulin treatment/ or insulin dependent diabetes mellitus/ or insulin/ (360982)
9 limit 8 to yr="2008 -Current" (185073)
10 insulin therapy.mp. or insulin treatment/ (16700)
11 limit 10 to yr="2008 -Current" (8835)
12 9 or 11 (185073)
13 haemoglobin.mp. or hemoglobin/ (79530)
14 limit 13 to yr="2008 -Current" (46243)
15 glycosylated hemoglobin/ or hemoglobin A1c/ or hemoglobin A/ or hemoglobin analysis/ or hemoglobin blood level/ or hemoglobin.mp. or hemoglobin/ (147579)
16 limit 15 to yr="2008 -Current" (86235)
17 HbA1c.mp. or hemoglobin A1c/ (42210)
18 limit 17 to yr="2008 -Current" (28330)
19 14 or 16 or 18 (88821)
20 5 and 7 and 12 and 19 (759)

****

Making inclusion/exclusion decisions.

Three reviewers will independently assess papers for inclusion/exclusion criteria using the title and articles' abstract. Disagreements will be resolved by discussion. Full paper copies of relevant or potentially relevant references will be obtained for detailed examination. Foreign language publications will be screened using English abstracts. Translations will be obtained where necessary or were possible, within the resources and timeframe of the project.

DATA HANDLING

Data extraction strategy

Data will be extracted using a pre-designed data extraction form, by one reviewer and checked by a two other reviewers. Where information is missing authors will be contacted, but within the resources and timeframe of the project. Data from studies with multiple publications will be extracted and reported as a single study, in case of discrepancies the publication with biggest representative population will be utilized.

Methods of analysis

A descriptive analysis of included studies will be undertaken and relevant evidence will be categorised and summarised in tables (excel and word). GLM model for regression analysis will be used since no intervention is assessed. When appropriate, weighted variable will be used e.g. GPD per capita, number of patients included into the study.

Identified research evidence will be appropriately interpreted according to the assessment of methodological strengths and weaknesses and the possibility of potential biases.

The following subgroup analyses will be undertaken:

- High-income countries' HbA_{1c} median values.
- Median value of HbA_{1c} with exclusion of measurements higher than 10%

Data extraction

Standard data extraction table designed for this study will be used.
General study characteristics: Abstract/Full-text article, Critical evaluation, Type of Study
Population: Country, GPD per capita, Number of patients included into the study, Age, T1DM duration
Control: Guideline HbA_{1c} targeted values
Outcomes: Primary; secondary; HbA_{1c} value, ΔHbA1c

TIMELINES

Meeting and Project Schedule

1st quarter of August 2013 – Presentation of the protocol and preliminary searches (scoping searches); allocation of work
Up to 26th August 2013 – Systematic search and screening by title and abstract:
September 2013 – Obtaining full-text papers
Till the end of May 2014 – assessment of eligibility (PIROS)
June – September 2014 – Data extraction [HbA_{1c}]
1st quarter of October 2014 – Search for guideline values
Till the end of November 2014 – e-mail contact with authors for data complementation
December 2014 – Data analysis
January 2015 – Conclusions and drafting the full-text article
End of January 2015 – Full-text article (supplementary data) submission.

Supplementary material 2. Search strategy examples.

Database: Embase <1996 to 2013 Week 34> Search Strategy:

----

1 paediatric.mp. or pediatrics/ (72936) 2
limit 1 to yr="2008 -Current" (39429) 3
pediatric.mp. or pediatrics/ (210372)

4 limit 3 to yr="2008 -Current" (116292)
5 2 or 4 (135620)
6 diabetes.mp. or diabetes mellitus/ (463736)
7 limit 6 to yr="2008 -Current" (259967)
8 insulin.mp. or insulin treatment/ or insulin dependent diabetes mellitus/ or insulin/ (360982)
9 limit 8 to yr="2008 -Current" (185073)
10 insulin therapy.mp. or insulin treatment/ (16700)
11 limit 10 to yr="2008 -Current" (8835)
12 9 or 11 (185073)
13 haemoglobin.mp. or hemoglobin/ (79530)
14 limit 13 to yr="2008 -Current" (46243)
15 glycosylated hemoglobin/ or hemoglobin A1c/ or hemoglobin A/ or hemoglobin analysis/ or hemoglobin blood level/ or hemoglobin.mp. or hemoglobin/ (147579)
16 limit 15 to yr="2008 -Current" (86235)
17 HbA1c.mp. or hemoglobin A1c/ (42210)
18 limit 17 to yr="2008 -Current" (28330)
19 14 or 16 or 18 (88821)
20 5 and 7 and 12 and 19 (759)

****
Database: Ovid MEDLINE(R) <1946 to
August Week 2 2013> Search Strategy:

----

1 Infant, Newborn/ or Infant/ or Child/ or Pediatrics/ or paediatric.mp. or Child, Preschool/ or Adolescent/ (2870946)

2 limit 1 to yr="2008 -Current" (580408)
3 pediatric.mp. or Pediatrics/ (187262)
4 limit 3 to yr="2008 -Current" (59566)
5 2 or 4 (586699)
6 Diabetes Mellitus, Type 1/ or diabetes.mp. (389842)
7 limit 6 to yr="2008 -Current" (125463)
8 Insulin/ or insulin therapy.mp. (161735)
9 limit 8 to yr="2008 -Current" (31546)
10 Hemoglobin A, Glycosylated/ or haemoglobin.mp. (45341)

11 limit 10 to yr="2008 -Current" (14104)

12 Hemoglobin A, Glycosylated/ or glycated.mp. (26259)

13 limit 12 to yr="2008 -Current" (10818)

14 11 or 13 (15239)

15 Hemoglobin A, Glycosylated/ or HbA1c.mp. or Hemoglobin A/ (30984)

16 limit 15 to yr="2008 -Current" (11949)

17 14 or 16 (17494)

18 5 and 7 and 9 and 17 (606)

****

Supplementary material 3. HbA$_{1c}$ comparisons within subgroups:

1. Comparison of actual HbA$_{1c}$ regarding targeted HbA$_{1c}$ level:

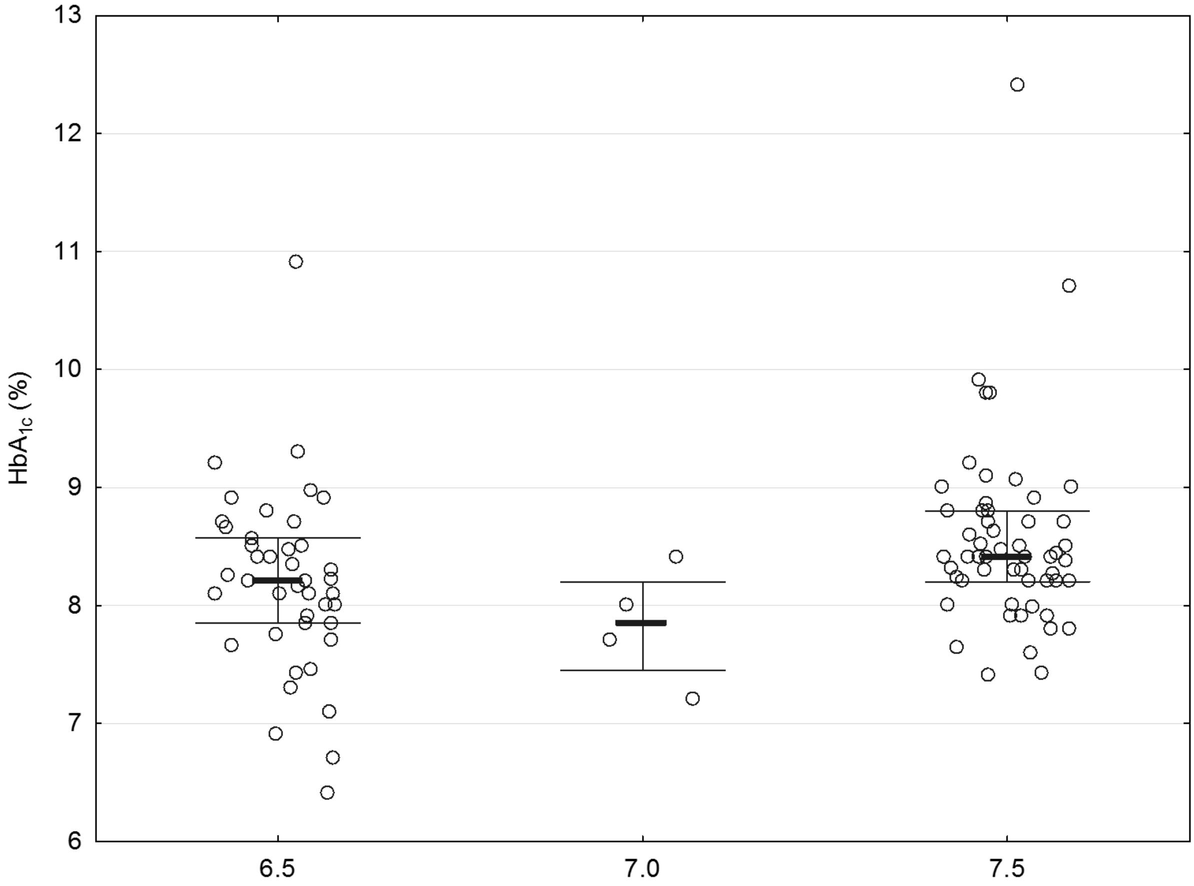

Figure 1 Comparison for actual HbA1c values between groups of 6.5% (47.53 mmol/mol), 7.0% (53 mmol/mol) and 7.5% (58.46 mmol/mol) as guideline values. (AKW p=0.0203)

Table 1 Comparison of actual HbA1c values regarding binding guideline levels. Values are given in % and mmol/mol in square brackets [mmol/mol]. (IQR – interquartile range, MWU – Mann-Whitney U-test, GLM – general linear model, AKW- KruskalWallis one-way analysis of variance)

Subgroup of:	Median (IQR) HbA$_{1c}$ in "6.5%"	Median (IQR) HbA$_{1c}$ in "7.5%"	p-value for comparison "6.5%" vs. "7.5%" (MWU; GLM with beta parameters)	Median (IQR) HbA$_{1c}$ in "7.0%"	p-value for comparison "6.5%" vs. "7.5%" vs. "7.0%" (AKW)
High-income countries	8.20 (7.858.67)%; [66.11 (62.2971.12) mmol/mol]	8.40 (8.208.70)%; [68.29 (66.1171.58) mmol/mol]	p=0.0935; p=0.0245, beta=-0.16	7.85 (7.7.458.20)%; [62.29 (57.91-66.11) mmol/mol]	0.0542

Studies without HbA$_{1c}$ values > 10%	8.20 (7.858.50)%; [66.11 (62.29-69.39) mmol/mol]	8.40 (8.208.70)%; [68.30 (66.1171.58) mmol/mol]	p=0.0287; p=0.0002, beta=-0.24	7.85 (7.458.20)%; [62.29 (57.91-66.11) mmol/mol]	0.0227

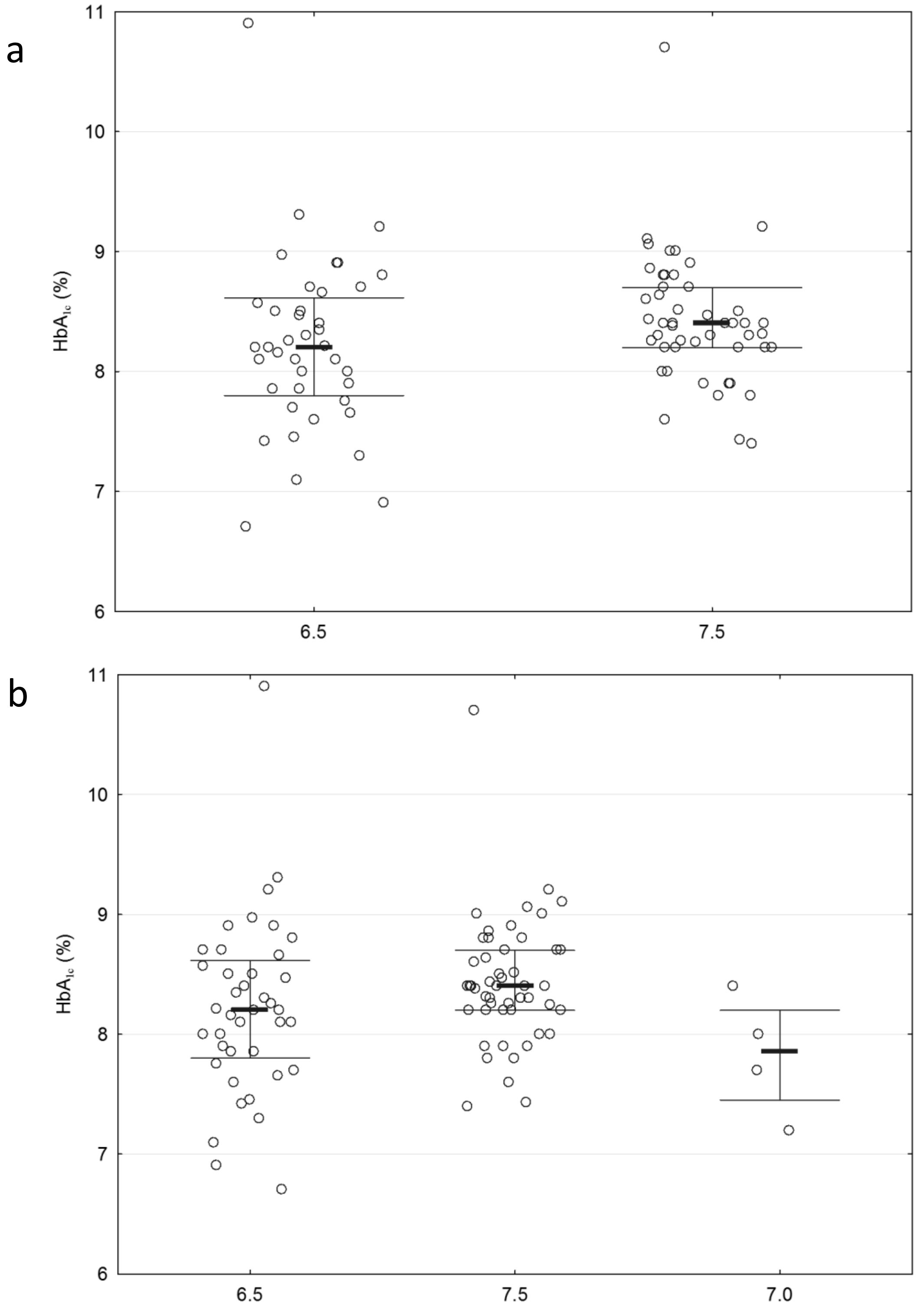

Figure 2 Comparison within high-income countries. "6.5" vs. "7.5" group **(a)** "6.5" vs. "7.0" vs. "7.5" **(b)**

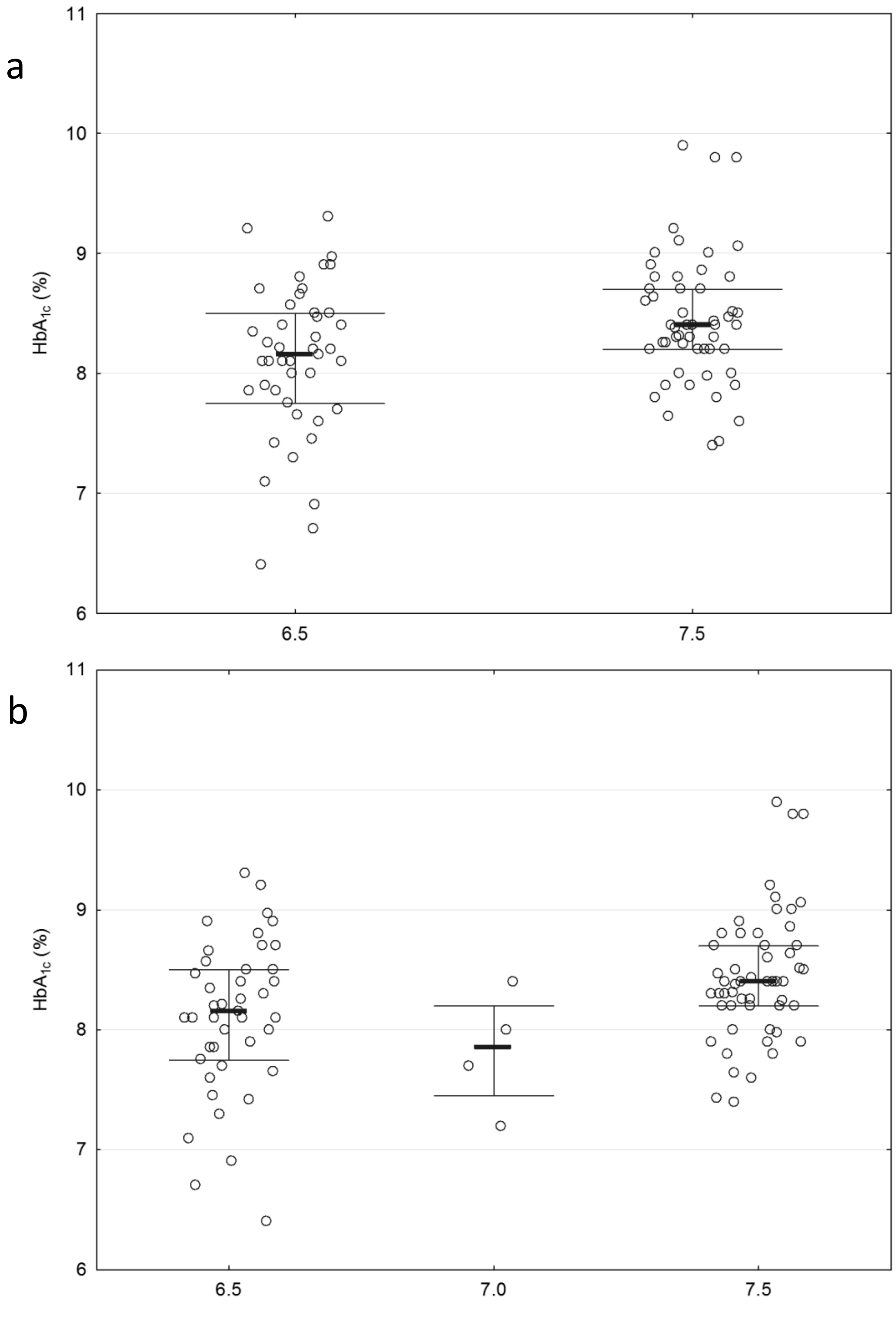

Figure 3 Comparison within studies without HbA1c values > 10%. "6.5" vs. "7.5" group **(a)** "6.5" vs. "7.0" vs. "7.5" **(b)**

2. Comparison of ΔHbA1c regarding targeted HbA1c level:

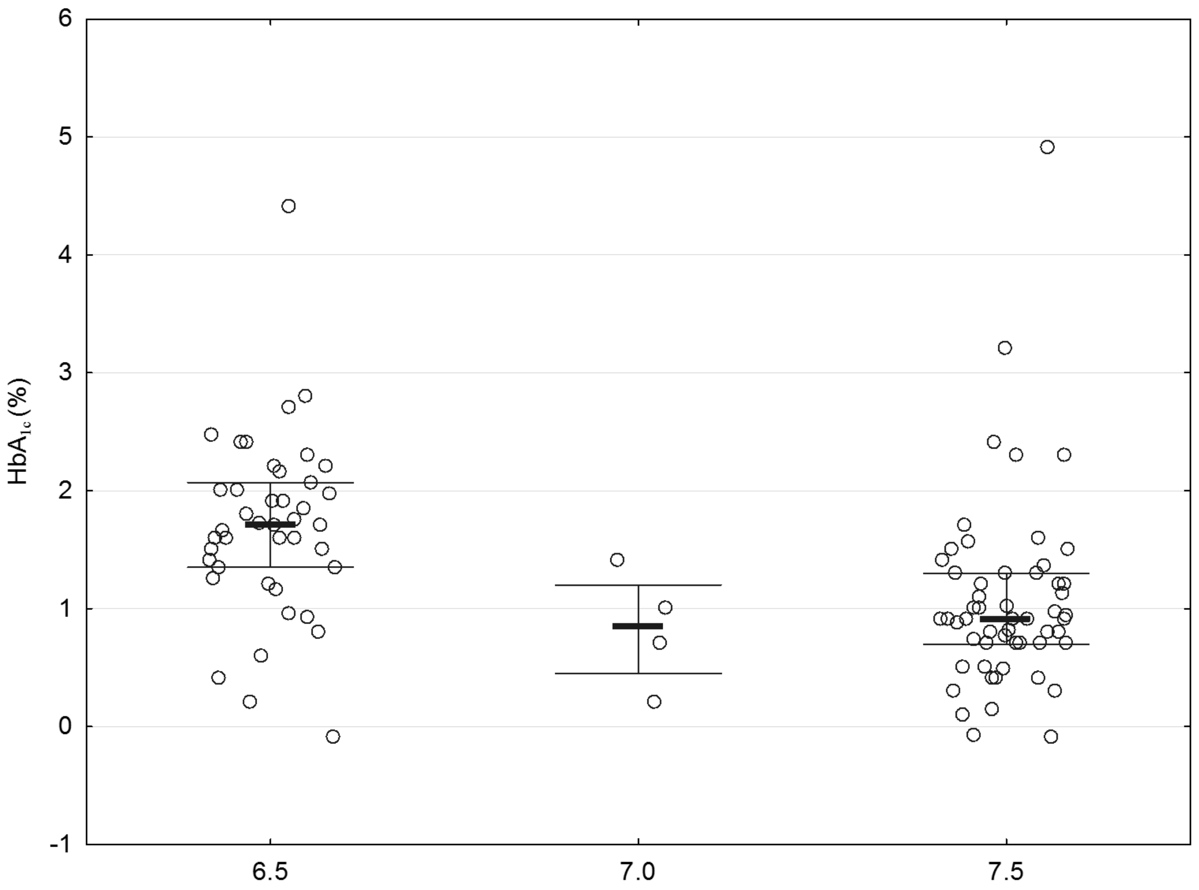

Figure 4 Comparison of ΔHbA1c regarding binding guideline values. (AKW, p=0.0001)

Table 2 Comparison of ΔHbA1c values regarding binding guideline levels. Values are given in % and mmol/mol in square brackets [mmol/mol]. (IQR – interquartile range, MWU – Mann-Whitney U-test, GLM – general linear model, AKW- KruskalWallis one-way analysis of variance)

Subgroup of:	Median (IQR) ΔHbA_{1c} in "6.5%"	Median (IQR) ΔHbA_{1c} in "7.5%"	p-value for comparison "6.5%" vs. "7.5%" (MWU; GLM with beta parameters)	Median (IQR) ΔHbA_{1c} in "7.0%"	p-value for comparison "6.5%" vs. "7.5%" vs. "7.0%" (AKW)
High-income countries	1.70 (1.352.00)%; [18.15 (13.12-21.86) mmol/mol]	0.90 (0.70-1.20)%; [8.19 (4.37-12.35) mmol/mol]	<0.0001	0.85 (0.45-1.20)%; [9.29 (4.92-13.12) mmol/mol]	<0.0001
Studies without HbA_{1c} values > 10%	1.70 (1.352.00)%; [18.58 (14.76-21.86) mmol/mol]	0.90 (0.70-1.20)%; [8.19 (4.37-12.35) mmol/mol]	<0.0001	0.85 (0.45-1.20)%; [9.29 (4.92-13.12) mmol/mol]	<0.0001

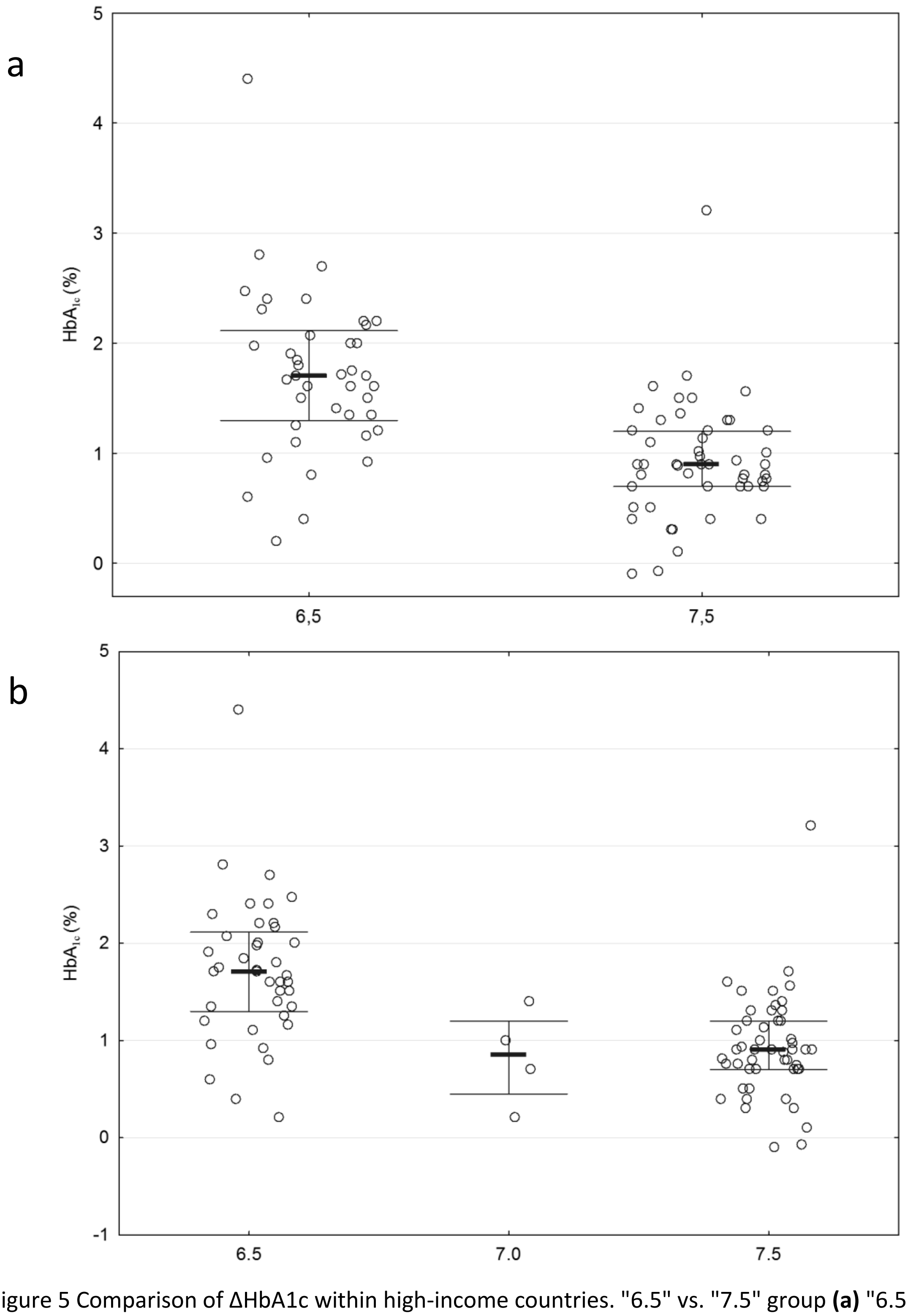

Figure 5 Comparison of ΔHbA1c within high-income countries. "6.5" vs. "7.5" group **(a)** "6.5" vs. "7.0" vs. "7.5" **(b)**

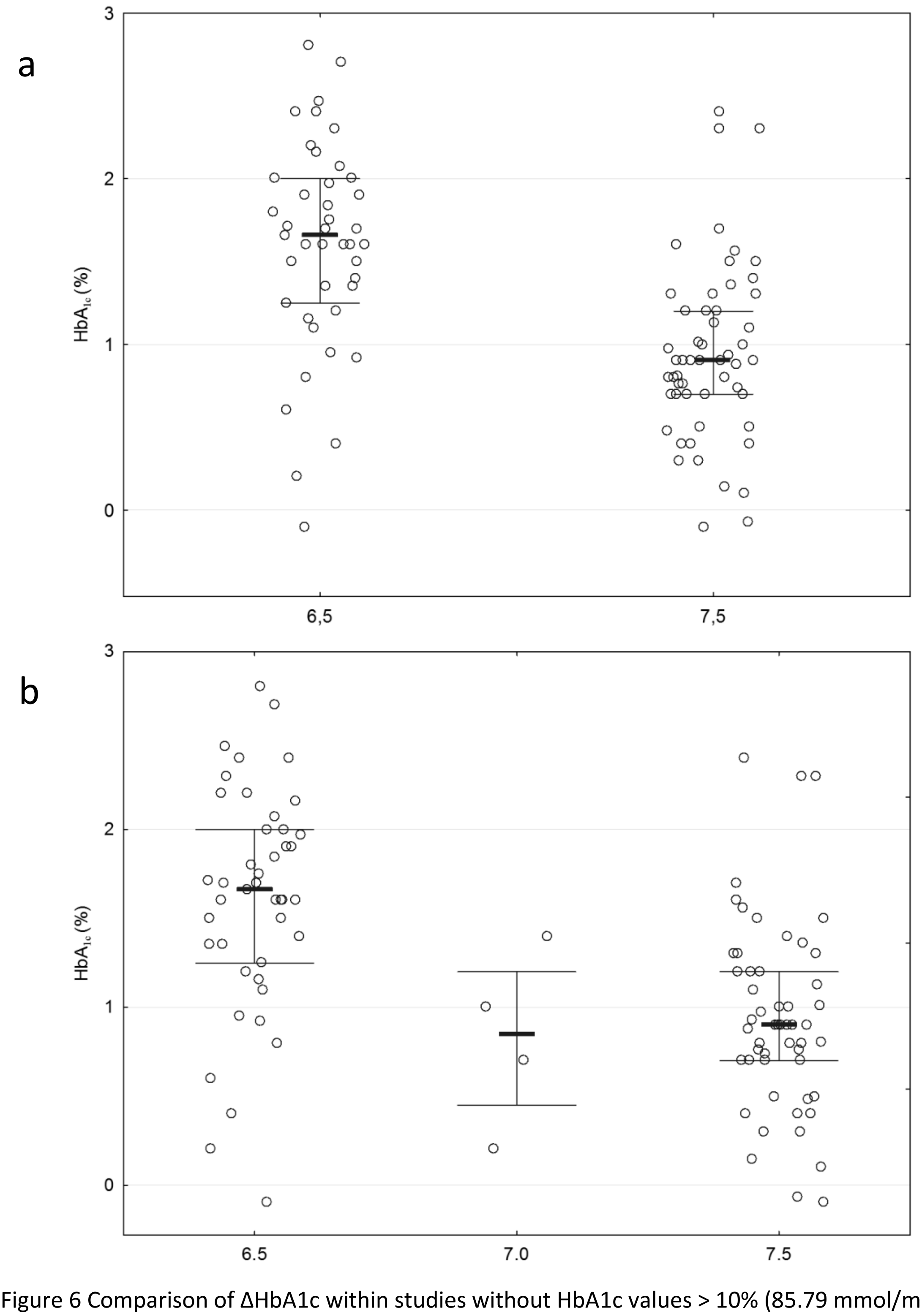

Figure 6 Comparison of ΔHbA1c within studies without HbA1c values > 10% (85.79 mmol/mol). "6.5" vs. "7.5" group **(a)** "6.5" vs. "7.0" vs. "7.5" **(b)**

Supplementary material 4. Forest plots of delta HbA_{1c}

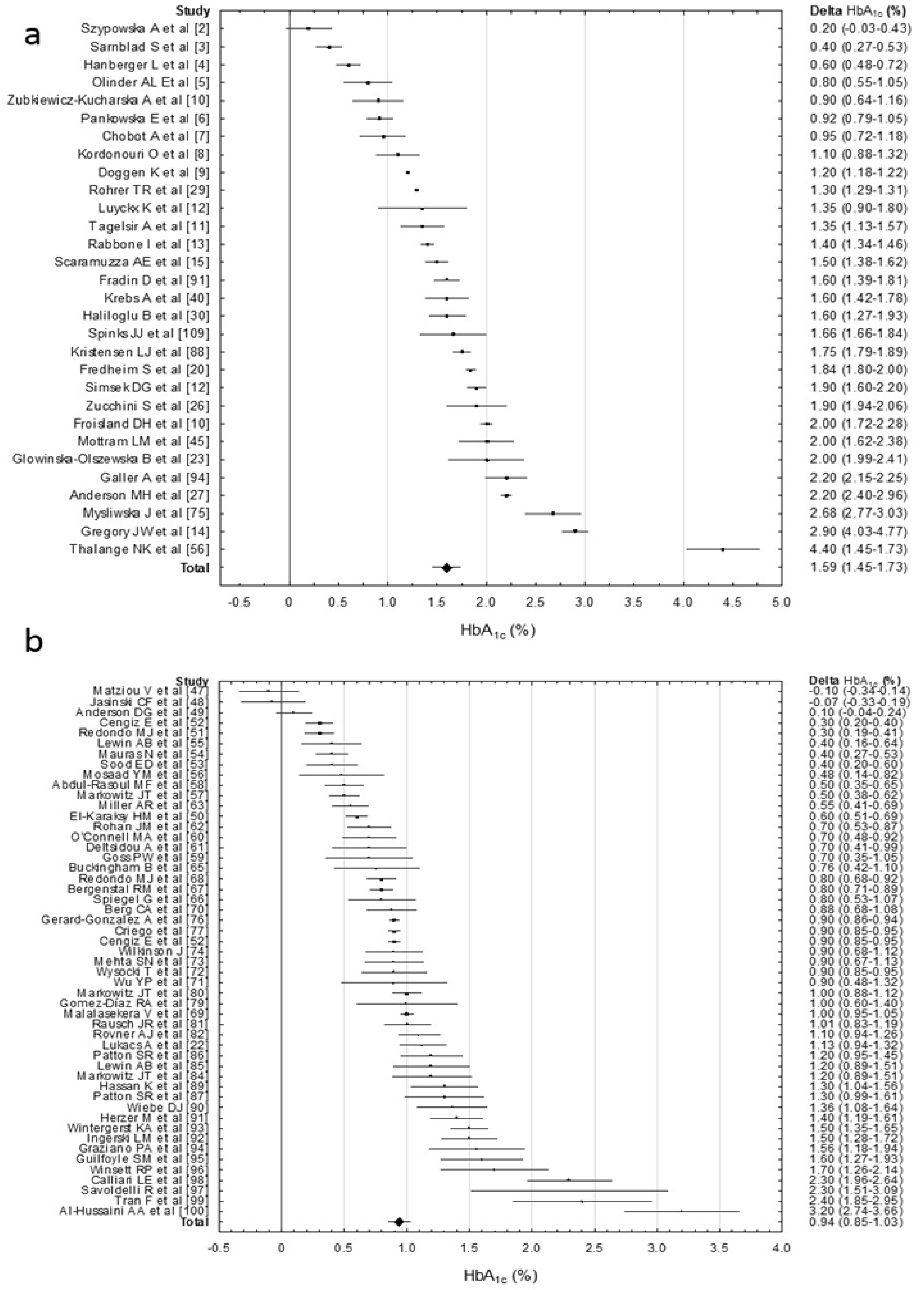

Figure 7 Forrest plot for difference between guideline and actual HbA_{1c}: A. among studies of 6.5% as the guideline value B. among studies of 7.5% as the guideline value

Supplementary material 5. Table with characteristics of included studies.

Characteristics of included studies with data extracted for quantitative analysis. Presented mean values concern the whole population of each study. Studies are presented in order of HbA_{1c} guideline, mean HbA_{1c} and study design. Regarding complications (diabetic ketoacidosis and hypoglycemia) “1” indicates that they occurred more frequently in the study population (specific subset of patients) than the literature reports [119, 120]. Null indicates in these columns on standard frequency. Regarding insulin therapy if more than a half of patients were treated with MDI then a study was appointed with “1” if more than 50% of patients were treated with CSII then a study was appointed with “0”. HbA1c – concentration of glycated hemoglobin A1c, GDP – gross domestic product, yrs. – years, DM – diabetes mellitus, MDI – multiple daily injections, CSII – continuous subcutaneous insulin infusion, NS – not stated in the paper

No.	Authors	Title	Journal article (1)/ conference proceeding (0)	Study design	Country	HbA_{1c} value according to local guidelines	Mean HbA_{1c} values in the study (%)	GDP per capita ($)	Number of patients in the study	Mean age in the study (yrs.)	Mean duration of DM in the study (yrs.)	Type of insulin therapy [MDI > 50% - 1; CSII > 50% - 0]	Hypoglycaemia in the beginning of the study (1 - more frequent than in the literature. 0 - normal)	Diabetic ketoacidosis in the beginning of the study (1 - more frequent than in the literature. 0 - normal)
1	Sumnik Z et al.	Long-term improvement of fasting glycaemia after switching basal insulin from NPH to detemir in children with type 1 diabetes: a 1-year multicentre study [85]	0	Cohort study	Czech Republic	6.5	6.40	18690	72	10.60	NS	0	NS	NS
2	Szypowska A. et al.	Insulin requirement in preschoolers treated with insulin pumps at onset of type 1 diabetes mellitus [111]	1	Case series	Poland	6.5	6.70	23273	58	3.3	1	NS	0	0
3	Sarnblad S et al.	Diabetes care in Swedish schools - A national survey [82]	0	Cross sectional	Sweden	6.5	6.90	55040	317	11.40	NS	NS	NS	NS
4	Hanberger L et al.	Health-related quality of life in intensively treated young	1	Cross sectional	Sweden	6.5	7.10	55040	400	13.20	5.10	1	NS	NS

		patients with type 1 diabetes [77]												
5	Olinder AL. Et al.	Missed bolus doses: devastating for metabolic control in CSII-treated adolescents with type 1 diabetes [110]	1	Cross sectional	Sweden	6.5	7.30	55040	90	14.80	7.90	0	NS	NS
6	Pankowska E et al.	Application of novel dual wave meal bolus and its impact on glycated haemoglobin A1c level in children with type 1 diabetes [78]	1	Cross sectional	Poland	6.5	7.42	23273	499	10.60	4.34	0	NS	NS
7	Chobot A et al.	Helicobacter pylori infection in type 1 diabetes children and adolescents using 13C urea breath test [28]	0	Cohort study	Poland	6.5	7.45	23273	129	13.30	4.43	NS	NS	NS
8	Kordonouri O et al.	Sensor augmented pump therapy from onset of type 1 diabetes: late follow-up results of the pediatric onset study [32]	1	Interventional study	Germany Austria, Switzerland	6.5	7.65	42597	131	NS	NS	0	NS	NS
9	Doggen K et al.	Care delivery and outcomes among Belgian children and adolescents with type 1 diabetes [24]	1	Cross sectional	Belgium	6.5	7.70	43399	974	12.70	4.30	1	1	0
10	Zubkiewicz-Kucharska A et al.	The efficacy of bolus calculator on metabolic control in pediatric patients using CSII [59]	0	Cross sectional	Poland	6.5	7.75	23273	129	NS	NS	NS	0	0
11	Tagelsir A et al.	Dental caries and dental care level (restorative index) in children with diabetes mellitus type 1 [46]	1	Case-control study	Belgium	6.5	7.85	43399	52	9.84	4.61	0	NS	NS

12	Luyckx K et al.	Glycemic control, coping, and internalizing and externalizing symptoms in adolescents with type 1 diabetes [103]	1	Cross sectional	Germany Austria, Switzerland	6.5	7.85	42597	109	13.77	4.95	NS	NS	NS
13	Rabbone I et al.	Pandemic influenza A H1N1 in Italian children and adolescents with type 1 diabetes [74]	0	Cross sectional	Italy	6.5	7.90	33816	1461	13.00	6.00	NS	NS	NS
14	Besser REJ et al.	Preserved endogenous insulin secretion as measured by urinary C-peptide creatinine ratio is associated with improved HbA1c and less glycaemic variability in paediatric Type 1 diabetes [42]	0	Cross sectional	UK	6.5	8.00	38920	135	13.20	3.90	NS	NS	NS
15	Scaramuzza AE et al.	Use of integrated real-time continuous glucose monitoring/insulin pump system in children and adolescents with type 1 diabetes: A 3-year follow-up study [54]	1	Cohort study	Italy	6.5	8.00	33816	622	13.02	6.22	0	0	0
16	Haliloglu B et al.	Diabetes related problems and diabetic controls among the school children with type 1 diabetes mellitus living in Istanbul [30]	0	Cohort study	Turkey	6.5	8.10	5480	114	NS	1.00	1	NS	NS
17	Haugstvedt A et al.	Fear of hypoglycemia in mothers and fathers of children with type 1 diabetes is associated with	1	Cross sectional	Norway	6.5	8.10	99636	114	10.60	3.90	NS	NS	NS

		poor glycaemic control and parental emotional distress: a population-based study [106]												
18	Krebs A et al.	Cardiovascular risk in pediatric type 1 diabetes: Sex-specific intima-media thickening verified by automatic contour identification and analyzing systems [40]	1	Cross sectional	Germany Austria, Switzerland	6.5	8.10	42597	270	13.75	5.70	NS	NS	NS
19	Fradin D et al.	Association of the CpG Methylation Pattern of the Proximal Insulin Gene Promoter with Type 1 Diabetes [91]	1	Case-control study	France	6.5	8.10	39746	485	12.10	7.50	NS	NS	NS
20	Spinks JJ et al.	Paediatric Diabetes services - evidence that expanding the workforce allows intensification of insulin regimens and improves glycaemic control [109]	1	Case series	UK	6.5	8.16	38920	70	NS	NS	NS	0	0
21	Huemer M et al.	Low levels of asymmetric dimethylarginine in children with diabetes mellitus type I compared with healthy children [55]	1	Cross sectional	Germany Austria, Switzerland	6.5	8.20	42597	85	12.30	4.08	NS	NS	NS
22	Cherubini V et al.	Metabolic control in Italian children with type 1 diabetes: Is it changing during the years? Preliminary results of vikids study [84]	0	Cross sectional	Italy	6.5	8.20	33816	792	NS	NS	NS	NS	NS

23	van Vliet M et al.	Overweight Is Highly Prevalent In Children with Type 1 Diabetes And Associates with Cardiometabolic Risk [72]	1	Cross sectional	Netherlands	6.5	8.22	45960	283	12.72	5.36	0	NS	NS
24	Kristensen LJ et al.	Psychometric Evaluation of the Adherence in Diabetes Questionnaire [88]	1	Cross sectional	Denmark	6.5	8.25	56364	766	12.30	5.20	NS	NS	NS
25	Skrivarhaug T et al.	Norwegian Childhood Diabetes Registry: Childhood onset diabetes in Norway 1973-2012 [16]	1	Cross sectional	Norway	6.5	8.30	99636	2520	NS	NS	0	0	1
26	Fredheim S et al.	Diabetic ketoacidosis at the onset of type 1 diabetes is associated with future HbA1c levels [20]	1	Cross sectional	Denmark	6.5	8.34	56364	2964	9.17	5.84	1	1	1
27	Zucchini S et al.	Usefulness of CGM with iPro2 in children with T1DM and correlations between Glucose Variability and metabolic control [26]	0	Cohort study	Italy	6.5	8.40	33816	70	13.80	7.40	1	NS	NS
28	Simsek DG et al.	Diabetes care, glycemic control, complications, and concomitant autoimmune diseases in children with type 1 diabetes in Turkey: A multicenter study [12]	1	Cohort study	Turkey	6.5	8.40	5480	1032	12.50	4.70	1	0	0
29	Rohrer TR et al.	Down's syndrome in diabetic patients aged <20 years: an analysis	1	Cross sectional	Germany Austria, Switzerland	6.5	8.47	42597	42281	13.81	5.44	NS	0	0

		of metabolic status, glycaemic control and autoimmunity in comparison with type 1 diabetes [104]												
30	Glowinska-Olszewska B et al.	Relationship between circulating endothelial progenitor cells and endothelial dysfunction in children with type 1 diabetes: a novel paradigm of early atherosclerosis in high-risk young patients [23]	1	Cohort study	Poland	6.5	8.50	23273	52	14.50	6.00	NS	NS	NS
31	Mottram LM et al.	Does physical activity and fitness influence glycaemic control and insulin requirement in children and young people with Type 1 diabetes? [45]	0	Cohort study	UK	6.5	8.50	38920	60	12.90	1.00	NS	NS	NS
32	Galler A et al.	Association Between Media Consumption Habits, Physical Activity, Socioeconomic Status, and Glycemic Control in Children, Adolescents, and Young Adults with Type 1 Diabetes [94]	1	Cross sectional	Germany Austria, Switzerland	6.5	8.57	42597	222	13.7	6.1	NS	NS	NS
33	Froisland DH et al.	Health-related quality of life among Norwegian children and adolescents with type 1 diabetes on intensive insulin treatment: a	1	Cohort study	Norway	6.5	8.66	99636	1952	13.83	5.43	0	0	1

		population-based study [10]												
34	Andersson MH et al.	Continuous glucose monitoring may improve metabolic control in children and adolescents with type 1 diabetes [27]	0	Cohort study	Sweden	6.5	8.70	55040	103	NS	NS	NS	NS	NS
35	Hughes CR et al.	Sustained benefits of continuous subcutaneous insulin infusion [92]	1	Case series	UK	6.5	8.70	38920	460	NS	NS	0	0	1
36	Dias R et al.	The effect of insulin intensification on glycaemic control and lipid levels in children and young persons with type 1 diabetes differs in relation to ethnic group [33]	0	Cross sectional	UK	6.5	8.80	38920	222	NS	NS	1	NS	NS
37	Hindmarsh PC et al.	Pediatric estimated average glucose from continuous glucose monitoring in children and young people with type 1 diabetes mellitus [80]	0	Cohort study	UK	6.5	8.90	38920	85	12.97	NS	NS	0	0
38	Hamersley S et al.	How many paediatric patients are making endogenous insulin? [43]	0	Cross sectional	UK	6.5	8.90	38920	137	13.20	NS	NS	NS	NS
39	Mysliwska J et al.	The -174GG interleukin-6 genotype is protective from retinopathy and nephropathy in juvenile onset type 1 diabetes mellitus [75]	1	Cohort study	Poland	6.5	8.97	23273	210	16.59	6.88	NS	0	0

40	Branco S et al.	Vitamin D deficiency in children and adolescents with type 1 diabetes [29]	0	Cohort study	Portugal	6.5	9.20	20175	68	NS	6.30	NS	NS	NS
41	Gregory JW et al.	Development and evaluation by a cluster randomised trial of a psychosocial intervention in children and teenagers experiencing diabetes: the DEPICTED study [14]	1	Interventional study	UK	6.5	9.30	38920	693	10.54	2.64	NS	NS	NS
42	Thalange NK et al.	Clinical experience with prandial biphasic insulin aspart 30/70 three-times daily (TID) in paediatric patients with type 1 diabetes (T1D): Results from a single-centre audit [56]	0	Cohort study	UK	6.5	10.90	38920	113	11.80	NS	1	NS	NS
43	Urakami T et al.	Association between sex, age, insulin regimens and glycemic control in children and adolescents with type 1 diabetes [69]	1	Cross sectional	Japan	7.0	7.20	46731	103	16.80	1.00	1	NS	NS
44	Urakami T et al.	Influence of plasma glucagon levels on glycemic control in children with type 1 diabetes [52]	1	Case series	Japan	7.0	7.70	46731	60	13.30	6.90	NS	NS	NS
45	Nakamura N et al.	Health-related and diabetes-related quality of life in Japanese children and adolescents with type 1 and type 2 diabetes [68]	1	Cross sectional	Japan	7.0	8.00	46731	368	14.00	6.50	NS	NS	NS

46	Barzel M et al.	Coparenting in Relation to Children's Psychosocial and Diabetes-Specific Adjustment [97]	1	Cross sectional	Canada	7.0	8.40	51206	61	10.70	4.90	NS	0	0
47	Matziou V et al.	Factors influencing the quality of life of young patients with diabetes [53]	1	Cohort study	Greece	7.5	7.40	22442	98	14.90	7.30	0	0	NS
48	Jasinski CF et al.	Healthcare cost of type 1 diabetes mellitus in new-onset children in a hospital compared to an outpatient setting [19]	1	Cross sectional	USA	7.5	7.43	51749	84	10.36	1.00	0	NS	NS
49	Anderson DG et al.	Multiple daily injections in young patients using the ezy-BICC bolus insulin calculation card, compared to mixed insulin and CSII [108]	1	Cohort study	Australia	7.5	7.60	67442	368	12.4	4.7	NS	0	0
50	El-Karaksy HM et al.	Prevalence of hepatic abnormalities in a cohort of Egyptian children with type 1 diabetes mellitus [66]	1	Cross sectional	Egypt	7.5	7.64	3256	692	10.48	3.91	1	NS	NS
51	Redondo MJ et al.	Characteristics of pediatric type 1 diabetes (T1D) that predict HbA1c at one year [38]	0	Cross sectional	USA	7.5	7.80	51749	654	10.20	1.00	NS	NS	1
52	Cengiz E et al.	How common are episodes of diabetic ketoacidosis (DKA) and severe hypoglycemia (SH) in the first year of diagnosis with type 1 diabetes (T1D)? [39]	0	Cohort study	USA	7.5	7.80	51749	795	9.20	1.00	NS	0	0

53	Sood ED et al.	Mother-father informant discrepancies regarding diabetes management: associations with diabetes-specific family conflict and glycemic control [13]	1	Cohort study	USA	7.5	7.90	51749	136	10.50	4.10	0	NS	NS
54	Mauras N et al.	A Randomized Clinical Trial to Assess the Efficacy and Safety of Real-Time Continuous Glucose Monitoring in the Management of Type 1 Diabetes in Young Children Aged 4 to 10 Years [93]	1	Interventional study	USA	7.5	7.90	51749	146	7.50	3.50	0	1	NS
55	Lewin AB et al.	Brief report: normative data on a structured interview for diabetes adherence in childhood [60]	1	Cohort study	USA	7.5	7.90	51749	275	13.30	2.90	1	0	NS
56	Mosaad YM et al.	HLA-DQB1* alleles and genetic susceptibility to type 1 diabetes mellitus [34]	1	Cross sectional	Egypt	7.5	7.98	3256	85	12.52	2.50	1	NS	0
57	Markowitz JT et al.	Re-examining a measure of diabetes-related burden in parents of young people with Type 1 diabetes: The Problem Areas in Diabetes Survey - Parent Revised version (PAID-PR) [41]	1	Cross sectional	USA	7.5	8.00	51749	376	12.90	6.30	0	NS	NS
58	M Abdul-Rasoul F et al.	Quality of Life of Children and Adolescents with	1	Cohort study	Kuwait	7.5	8.00	56374	436	9.10	5.37	1	NS	NS

		Type 1 Diabetes in Kuwait [15]												
59	Goss PW et al.	A 'radical' new rural model for pediatric diabetes care [62]	1	Cohort study	Australia	7.5	8.20	67442	61	13.90	NS	NS	NS	NS
60	O'Connell MA et al.	Poor adherence to integral daily tasks limits the efficacy of CSII in youth [49]	1	Case series	Australia	7.5	8.20	67442	100	13.60	6.10	NS	NS	NS
61	Deltsidou A et al.	Age at Menarche and Menstrual Irregularities of Adolescents with Type 1 Diabetes [63]	1	Case-control study	Greece	7.5	8.20	22442	100	15.00	NS	0	NS	NS
62	Rohan JM et al.	Identification of self-management patterns in pediatric type 1 diabetes using cluster analysis [50]	1	Cross sectional	USA	7.5	8.20	51749	239	10.54	4.41	1	NS	NS
63	Miller AR et al.	Insulin dose changes in children attending a residential diabetic camp [98]	1	Cross sectional	USA	7.5	8.20	51749	256	11.60	4.67	0	0	NS
64	Pingul MM et al.	Pediatric diabetes outpatient center at Rhode Island hospital: The impact of changing initial diabetes education from inpatient to outpatient [44]	0	Cohort study	USA	7.5	8.24	51749	152	10.60	1.00	NS	NS	NS
65	Buckingham B et al.	Effectiveness and safety study of the prototype 4th generation seven day continuous glucose monitoring system in youth with type 1 diabetes mellitus [58]	0	Interventional study	USA	7.5	8.26	51749	72	12.60	6.30	0	NS	NS

66	Spiegel G et al.	Randomized Nutrition Education Intervention to Improve Carbohydrate Counting in Adolescents with Type 1 Diabetes Study: Is More Intensive Education Needed? [113]	1	Interventional study	USA	7.5	8.30	51749	66	15.10	5.50	0	NS	NS
67	Bergenstal RM et al.	Effectiveness of sensor-augmented insulin-pump therapy in type 1 diabetes [101]	1	Interventional study	USA	7.5	8.30	51749	156	12.2	5.05	1	0	0
68	Redondo MJ et al.	Types of pediatric diabetes mellitus defined by anti-islet autoimmunity and random C-peptide at diagnosis [11]	1	Cohort study	USA	7.5	8.30	51749	607	10.20	2.00	NS	NS	1
69	Malalasekera V et al.	Potential reno-protective effects of a gluten-free diet in type 1 diabetes [79]	1	Cross sectional	Australia	7.5	8.31	67442	59	14.19	7.06	NS	NS	NS
70	Berg CA et al.	Parental Involvement and Adolescents' Diabetes Management: The Mediating Role of Self-Efficacy and Externalizing and Internalizing Behaviors [112]	1	Cross sectional	USA	7.5	8.38	51749	252	12.49	1.00	0	NS	NS
71	Wu YP et al.	Is insulin pump therapy better than injection for adolescents with diabetes? [64]	1	Cohort study	USA	7.5	8.40	51749	62	14.20	NS	1	NS	NS
72	Wysocki T et al.	Diabetes Problem Solving by Youths with Type 1 Diabetes and their Caregivers: Measurement,	1	Cohort study	USA	7.5	8.40	51749	114	12.10	5.80	NS	NS	NS

		Validation and Longitudinal Associations with Glycemic Control [86]												
73	Mehta SN et al.	Dietary Behaviors Predict Glycemic Control in youth with type 1 diabetes [114]	1	Cross sectional	USA	7.5	8.40	51749	119	12.10	5.40	1	NS	NS
74	Wilkinson J	Factors affecting improved glycaemic control in youth using insulin pumps [100]	1	Cross sectional	USA	7.5	8.40	51749	150	13.6	7.1	NS	0	0
75	Cengiz E et al.	Resetting the bar: Frequency of severe hypoglycemia (SH) and diabetic ketoacidosis (DKA) among children with type 1 diabetes (T1D) in the T1D exchange registry [37]	0	Cohort study	USA	7.5	8.40	51749	4120	11.90	5.20	NS	0	1
76	Gerard-Gonzalez A et al.	Comparison of autoantibody-positive and autoantibody-negative pediatric participants enrolled in the T1D Exchange clinic registry [17]	1	Cross sectional	USA	7.5	8.40	51749	6737	7.80	NS	NS	NS	NS
77	Criego et al.	Increased Body Mass Index (BMI) is associated with higher hemoglobin A1c (A1c) among 6-12 year olds but is not associated with total daily insulin dose per kg (TDI) in type 1 diabetes (T1D) participants enrolled in T1D Exchange Clinic Registry [57]	0	Cross sectional	USA	7.5	8.43	51749	4427	12.90	5.80	NS	0	0

78	Lawrence JM et al.	Diabetes-related quality of life and glycaemic control among youth with type 1 diabetes [81]	0	Cross sectional	USA	7.5	8.47	51749	2601	13.60	5.20	NS	0	0
79	Gomez-Díaz RA et al	Association between carotid intima-media thickness, buccodental status, and glycemic control in pediatric type 1 diabetes [31]	1	Cross sectional	Mexico	7.5	8.50	9749	69	11.60	5.10	NS	NS	NS
80	Markowitz JT et al.	Validation of an abbreviated adherence measure for young people with Type1 diabetes [47]	1	Cohort study	USA	7.5	8.50	51749	338	12.50	5.40	1	NS	NS
81	Rausch JR et al.	Changes in Treatment Adherence and Glycemic Control During the Transition to Adolescence in Type 1 Diabetes [90]	1	Cohort study	USA	7.5	8.51	51749	225	12.62	6.46	0	NS	NS
82	Rovner AJ et al.	Development and validation of the type 1 diabetes nutrition knowledge survey [36]	1	Cohort study	USA	7.5	8.60	51749	282	13.30	6.40	0	NS	NS
83	Lukacs A et al.	Benefits of continuous subcutaneous insulin infusion on quality of life [22]	1	Cross sectional	Hungary	7.5	8.63	23236	239	13.36	6.03	1	1	NS
84	Markowitz JT et al.	Brief screening tool for disordered eating in diabetes: Internal consistency and external validity in a contemporary sample of pediatric patients	1	Cross sectional	USA	7.5	8.70	51749	112	15.10	6.80	1	NS	NS

		with type 1 diabetes [70]												
85	Lewin AB et al.	Validity and reliability of an adolescent and parent rating scale of type 1 diabetes adherence behaviors: The self-care inventory (SCI) [76]	1	Cohort study	USA	7.5	8.70	51749	164	14.60	4.70	0	NS	NS
86	Patton SR et al.	Survey of Insulin Site Rotation in Youth With Type 1 Diabetes Mellitus [61]	1	Cohort study	USA	7.5	8.70	51749	201	11.80	5.90	1	NS	NS
87	Patton SR et al.	Frequency of Mealtime Insulin Bolus as a Proxy Measure of Adherence for Children and Youths with Type 1 Diabetes Mellitus [87]	1	Interventional study	USA	7.5	8.80	51749	100	12.70	1.00	0	NS	NS
88	Cortina S et al.	Sociodemographic and psychosocial factors associated with continuous subcutaneous insulin infusion in adolescents with type 1 diabetes [102]	1	Cohort study	USA	7.5	8.80	51749	150	15.47	6.04	0	NS	NS
89	Hassan K et al.	Glycemic control in pediatric type 1 diabetes: Role of caregiver literacy [67]	1	Cross sectional	USA	7.5	8.80	51749	200	11.80	4.80	1	NS	NS
90	Wiebe DJ	Longitudinal Associations of Maternal Depressive Symptoms, Maternal Involvement, and Diabetes Management Across Adolescence [96]	1	Cross sectional	USA	7.5	8.86	51749	82	12.79	NS	NS	NS	NS

91	Herzer MH et al.	Anxiety symptoms in adolescents with type 1 diabetes: association with Blood Glucose Monitoring and glycemic control [105]	1	Cross sectional	USA	7.5	8.90	51749	276	15.60	6.60	0	NS	NS
92	Ingerski LM et al.	Correlates of glycemic control and quality of life outcomes in adolescents with type 1 diabetes [65]	1	Cohort study	USA	7.5	9.00	51749	261	15.70	7.00	0	NS	NS
93	Wintergerst KA et al.	The impact of health insurance coverage on pediatric diabetes management [71]	1	Cross sectional	USA	7.5	9.00	51749	701	13.50	NS	1	NS	NS
94	Graziano PA et al.	Gender differences in the relationship between parental report of self-regulation skills and adolescents' management of type 1 diabetes [51]	1	Cohort study	USA	7.5	9.06	51749	109	15.23	5.06	NS	NS	NS
95	Guilfoyle SM et al.	Blood glucose monitoring and glycemic control in adolescents with type 1 diabetes: meter downloads versus self-report [95]	1	Cohort study	USA	7.5	9.10	51749	143	16.00	6.50	0	NS	NS
96	Winsett RP et al.	Adolescent self-efficacy and resilience in participants attending A diabetes camp [99]	1	Cohort study	USA	7.5	9.20	51749	81	13.40	6.63	0	NS	NS
97	Savoldelli R et al.	Vitamin D insufficiency in a Brazilian type 1 diabetes mellitus pediatric population [73]	0	Case series	Brazil	7.5	9.80	14987	117	NS	NS	NS	0	0

98	Calliari LE et al.	Ten year evolution on diagnosis and treatment of type 1 diabetes mellitus in an university center in Sao Paulo, Brazil [83]	0	Cohort study	Brazil	7.5	9.80	14987	132	13.3	6.50	NS	0	NS
99	Tran F et al.	Glycaemic control in children with neonatal diabetes and type 1 diabetes in Vietnam [48]	1	Cross sectional	Vietnam	7.5	9.90	1755	93	11.50	2.60	1	0	NS
100	Al-Hussaini AA et al.	Is There an Association between Type 1 Diabetes in Children and Gallbladder Stones Formation? [21]	1	Cohort study	Saudi Arabia	7.5	10.70	25136	105	8.50	2.20	NS	NS	NS
101	Mukama LJ et al.	Improved glycemic control and acute complications among children with type 1 diabetes mellitus in Moshi, Tanzania [18]	1	Cross sectional	Tanzania	7.5	12.40	609	81	NS	1.00	1	0	1
102	Juvenile Diabetes Research Foundation Continuous Glucose Monitoring Study Group	Effectiveness of continuous glucose monitoring in a clinical care environment [107]	1	Interventional study	International	International	7.80	International	50	NS	NS	NS	1	NS
103	Phillip M et al.	Nocturnal Glucose Control with an Artificial Pancreas at a Diabetes Camp [89]	1	Interventional study	International	International	8.00	International	56	13.8	7	0	0	0
104	de Wit M et al.	Assessing diabetes-related quality of life of youth with type 1 diabetes in routine clinical	1	Cohort study	International	International	8.10	International	84	14.40	6.40	NS	NS	NS

		care: the MIND Youth Questionnaire (MY-Q) [25]												
105	Adolfsson P et al.	Safety and patient perception of an insulin pen with simple memory function for children and adolescents with type 1 diabetes the REMIND study [35]	1	Cohort study	International	International	8.40	International	354	12.00	3.80	1	NS	NS

[1] Sumnik Z VJ, Brazdova L, Skvor J. Long-term improvement of fasting glycaemia after switching basal insulin from NPH to determir in children with type 1 diabetes: a 1-year multicentre study. Casopis lekaru ceskych 2009; 147 (8): 421-425

[2] Szypowska A, Lipka M, Blazik M, Groele L, Pankowska E. Insulin requirement in preschoolers treated with insulin pumps at the onset of type 1 diabetes mellitus. Acta paediatrica 2009; 98: 527-530

[3] Sarnblad S BL, Detlofsson I, Jonsson A, Forsander G. Diabetes care in Swedish schools - A national survey. Pediatric diabetes 2009; 10: 67-68

[4] Hanberger L, Ludvigsson J, Nordfeldt S. Health-related quality of life in intensively treated young patients with type 1 diabetes. Pediatric diabetes 2009; 10: 374-381

[5] Olinder AL, Kernell A, Smide B. Missed bolus doses: devastating for metabolic control in CSII-treated adolescents with type 1 diabetes. Pediatric diabetes 2009; 10: 142-148

[6] Pankowska E, Szypowska A, Lipka M, Szpotanska M, Blazik M, Groele L. Application of novel dual wave meal bolus and its impact on glycated hemoglobin A1c level in children with type 1 diabetes. Pediatric diabetes 2009; 10: 298-303

[7] Chobot A, Skala-Zamorowska E, Bak-Drabik K Krzywicka A, Kwiecien J, Polanska J. Helicobacter pylori infection in type 1 diabetes children and adolescents using 13C urea breath test. Pediatric diabetes 2012; 13: 99

[8] Kordonouri O, Hartmann R, Pankowska E et al. Sensor augmented pump therapy from onset of type 1 diabetes: late follow-up results of the Pediatric Onset Study. Pediatric diabetes 2012; 13: 515-518

[9] Doggen K, Debacker N, Beckers D et al. Care delivery and outcomes among Belgian children and adolescents with type 1 diabetes. European journal of pediatrics 2012; 171: 1679-1685

[10] Zubkiewicz-Kucharska A SB, Chrzanowska J, Noczynska A. The efficacy of bolus calculator on metabolic control in pediatric patients using CSII. . Diabetes Technology and Therapeutics 2011; 13 [Suppl. 2]: 286

[11] Tagelsir A, Cauwels R, van Aken S, Vanobbergen J, Martens LC. Dental caries and dental care level (restorative index) in children with diabetes mellitus type 1. International journal of pediatric dentistry / the British Paedodontic Society [and] the International Association 2011; 21:13-22

[12] Luyckx K, Seiffge-Krenke I, Hampson SE. Glycemic control, coping, and internalizing and externalizing symptoms in adolescents with type 1 diabetes: a cross-lagged longitudinal approach. Diabetes care 2010; 33: 1424-1429

[13] Rabbone I SA, Iafusco D, Bonfanti R et al. Pandemic influenza A H1N1 in Italian children and adolescents with type 1 diabetes. Pediatric diabetes 2010; 11 [Suppl. 14]: 49

[14] Besser REJ CP, Shields BM, McDonald TJ, Jones AG, Knight BA, Hattersley AT. Preserved endogenous insulin secretion as measured by urinary C-peptide creatinine ratio is associated with improved HbA1c and less glycaemic variability in pediatric Type 1 diabetes. Diabetic Medicine 2012; 29 [Supp. 1]: 88

[15] Scaramuzza AE, Iafusco D, Rabbone I et al. Use of integrated real-time continuous glucose monitoring/insulin pump system in children and adolescents with type 1 diabetes: a 3-year follow-up study. Diabetes technology & therapeutics 2011; 13: 99-103

[16] Haliloglu B AE, Atay Z, Guran T, Bereket A, Turan S. Diabetes related problems and diabetic controls among the school children with type 1 diabetes mellitus living in Istanbul. Pediatric diabetes 2012; 13 [Suppl 17]: 1-173

[17] Haugstvedt A, Wentzel-Larsen T, Graue M, Sovik O, Rokne B. Fear of hypoglycemia in mothers and fathers of children with Type 1 diabetes is associated with poor glycaemic control and parental emotional distress: a population-based study. Diabetic medicine: a journal of the British Diabetic Association 2010; 27: 72-78

[18] Krebs A, Schmidt-Trucksass A, Doerfer J et al. Cardiovascular risk in pediatric type 1 diabetes: sex-specific intima-media thickening verified by automatic contour identification and analyzing systems. Pediatric diabetes 2012; 13: 251-258

[19] Galler A, Lindau M, Ernert A, Thalemann R, Raile K. Associations between media consumption habits, physical activity, socioeconomic status, and glycemic control in children, adolescents, and young adults with type 1 diabetes. Diabetes care 2011; 34: 2356-2359

[20] Spinks JJ, Haest J, Ross K, London R, Edge JA. Paediatric Diabetes Services--evidence that expanding the workforce allows intensification of insulin regimens and improves glycaemic control. Archives of disease in childhood 2009; 94: 646-647

[21] Huemer M, Simma B, Mayr D et al. Low levels of asymmetric dimethylarginine in children with diabetes mellitus type I compared with healthy children. The Journal of pediatrics 2011; 158: 602-606

[22] Cherubini V GR, Mosca A, Bonfanti R et al. Metabolic control in Italian children with type 1 diabetes: Is it changing during the years? Preliminary results of vikids study. Pediatric diabetes 2009; 10: 20

[23] van Vliet M, Van der Heyden JC, Diamant M et al. Overweight is highly prevalent in children with type 1 diabetes and associates with cardiometabolic risk. The Journal of pediatrics 2010; 156: 923-929

[24] Kristensen LJ, Thastum M, Mose AH, Birkebaek NH. Psychometric evaluation of the adherence in diabetes questionnaire. Diabetes care 2012; 35: 2161-2166

[25] Skrivarhaug T. Norwegian Childhood Diabetes Registry: Childhood onset diabetes in Norway 1973-2012. Norsk Epidemiologi 2013; 23: 23-27

[26] Fredheim S, Johannesen J, Johansen A et al. Diabetic ketoacidosis at the onset of type 1 diabetes is associated with future HbA1c levels. Diabetologia 2013; 56: 995-1003

[27] Zucchini S SM, Predieri B, Iughetti L et al. Usefulness of CGM with iPro2 in children with T1DM and correlations between Glucose Variability and metabolic control. . Pediatric diabetes 2012; 13

[28] Simsek DG, Aycan Z, Ozen S, et al. Diabetes care, glycemic control, complications, and concomitant autoimmune diseases in children with type 1 diabetes in Turkey: a multicenter study. Journal of clinical research in pediatric endocrinology 2013; 5: 20-26

[29] Rohrer TR, Hennes P, Thon A et al. Down's syndrome in diabetic patients aged <20 years: an analysis of metabolic status, glycaemic control and autoimmunity in comparison with type 1 diabetes. Diabetologia 2010; 53: 1070-1075

[30] Glowinska-Olszewska B, Moniuszko M, Hryniewicz A et al. Relationship between circulating endothelial progenitor cells and endothelial dysfunction in children with type 1 diabetes: a novel paradigm of early atherosclerosis in high-risk young patients. European journal of endocrinology / European Federation of Endocrine Societies 2013; 168: 153-161

[31] Mottram LM JR, Shield JPH, Burren CP. Does physical activity and fitness influence glycaemic control and insulin requirement in children and young people with Type 1 diabetes? Diabetic Medicine 2011; 47

[32] Urakami T, Suzuki J, Yoshida A et al. Association between Sex, Age, Insulin Regimens and Glycemic Control in Children and Adolescents with Type 1 Diabetes. Clinical pediatric endocrinology: case reports and clinical investigations: official journal of the Japanese Society for Pediatric Endocrinology 2010; 19: 1-6

[33] Froisland DH, Graue M, Markestad T, Skrivarhaug T, Wentzel-Larsen T, Dahl-Jorgensen K. Health-related quality of life among Norwegian children and adolescents with type 1 diabetes on intensive insulin treatment: a population-based study. Acta paediatrica 2013

[34] Andersson MH L-OM, Carlsson A. Continuous glucose monitoring may improve metabolic control in children and adolescents with type 1 diabetes. Pediatric diabetes 2012; 13

[35] Hughes CR, McDowell N, Cody D, Costigan C. Sustained benefits of continuous subcutaneous insulin infusion. Archives of disease in childhood 2012; 97: 245-247

[36] Dias R BF, Wyatt C, Cheema S, Allgrove J, Amin R. The effect of insulin intensification on glycaemic control and lipid levels in children and young persons with type 1 diabetes differs in relation to ethnic group. Hormone Research in Pediatrics 2012; 78: 58-59

[37] Hindmarsh PC. Pediatric estimated average glucose from continuous glucose monitoring in children and young people with type 1 diabetes mellitus. Diabetes 2009; 58

[38] Hamersley S SM, Besser REJ, McDonald TJ, Hattersley AT. How many pediatric patients are making endogenous insulin? Diabetic Medicine 2012; 29

[39] Mysliwska J, Zorena K, Mysliwiec M, Malinowska E, Raczynska K, Balcerska A. The -174GG interleukin-6 genotype is protective from retinopathy and nephropathy in juvenile onset type 1 diabetes mellitus. Pediatric research 2009; 66: 341-345

[40] Branco S RH, Costa C, Correia C, Fontoura M. Vitamin D deficiency in children and adolescents with type 1 diabetes. Pediatric diabetes 2012; 13

[41] Gregory J, Robling M, Bennert K et al. Development and evaluation by a cluster randomised trial of a psychosocial intervention in children and teenagers experiencing diabetes: the DEPICTED study. Health technology assessment 2011; 15: 1-202

[42] Thalange NK L-WH, Datta V, Stella P. Clinical experience with prandial biphasic insulin aspart 30/70 three-times daily (TID) in paediatric patients with type 1 diabetes (T1D): Results from a single-centre audit. Pediatric Diabetes 2011; 12: 124

[43] Fradin D, Le Fur S, Mille C et al. Association of the CpG methylation pattern of the proximal insulin gene promoter with type 1 diabetes. PloS one 2012; 7: e36278

[44] Urakami T, Nagano N, Suzuki J, Yoshida A, Takahashi S, Mugishima H. Influence of plasma glucagon levels on glycemic control in children with type 1 diabetes. Pediatrics international: official journal of the Japan Pediatric Society 2011; 53: 46-49

[45] Nakamura N, Sasaki N, Kida K, Matsuura N. Health-related and diabetes-related quality of life in Japanese children and adolescents with type 1 and type 2 diabetes. Pediatrics international: official journal of the Japan Pediatric Society 2010; 52: 224-229

[46] Barzel M, Reid GJ. Coparenting in relation to children's psychosocial and diabetes-specific adjustment. Journal of pediatric psychology 2011; 36: 618-629

[47] Matziou V, Tsoumakas K, Vlahioti E et al. Factors influencing the quality of life of young patients with diabetes. Journal of diabetes 2011; 3: 82-90

[48] Jasinski CF, Rodriguez-Monguio R, Tonyushkina K, Allen H. Healthcare cost of type 1 diabetes mellitus in new-onset children in a hospital compared to an outpatient setting. BMC pediatrics 2013; 13: 55

[49] Anderson DG. Multiple daily injections in young patients using the ezy-BICC bolus insulin calculation card, compared to mixed insulin and CSII. Pediatric diabetes 2009; 10: 304-309

[50] El-Karaksy HM, Anwar G, Esmat G et al. Prevalence of hepatic abnormalities in a cohort of Egyptian children with type 1 diabetes mellitus. Pediatric diabetes 2010; 11: 462-470

[51] Redondo MJ W-JS, Buckingham B, Kollman C et al. Characteristics of pediatric type 1 diabetes (T1D) that predict HbA1c at one year. Diabetes 2012; 61: A329-A330

[52] Cengiz E W-JS, Kollman C, Haymond M, Klingensmith G, Lee J, Tamborlane W. How common are episodes of diabetic ketoacidosis (DKA) and severe hypoglycemia (SH) in the first year of diagnosis with type 1 diabetes (T1D)? Diabetes 2012; 61: A328-A329

[53] Sood ED, Pendley JS, Delamater AM, Rohan JM, Pulgaron ER, Drotar D. Mother-father informant discrepancies regarding diabetes management: associations with diabetes-specific family conflict and glycemic control. Health psychology: official journal of the Division of Health Psychology, American Psychological Association 2012; 31: 571-579

[54] Mauras N, Beck R, Xing D et al. A randomized clinical trial to assess the efficacy and safety of real-time continuous glucose monitoring in the management of type 1 diabetes in young children aged 4 to <10 years. Diabetes care 2012; 35: 204-210

[55] Lewin AB, Storch EA, Williams LB, Duke DC, Silverstein JH, Geffken GR. Brief report: normative data on a structured interview for diabetes adherence in childhood. Journal of pediatric psychology 2010; 35: 177-182

[56] Mosaad YM, Auf FA, Metwally SS et al. HLA-DQB1* alleles and genetic susceptibility to type 1 diabetes mellitus. World journal of diabetes 2012; 3: 149-155

[57] Markowitz JT, Volkening LK, Butler DA, Antisdel-Lomaglio J, Anderson BJ, Laffel LM. Re-examining a measure of diabetes-related burden in parents of young people with Type 1 diabetes: the Problem Areas in Diabetes Survey - Parent Revised version (PAID-PR). Diabetic medicine: a journal of the British Diabetic Association 2012; 29: 526-530

[58] Abdul-Rasoul M, AlOtaibi F, Abdulla A, Rahme Z, AlShawaf F. Quality of life of children and adolescents with type 1 diabetes in Kuwait. Medical principles and practice: international journal of the Kuwait University, Health Science Centre 2013; 22: 379-384

[59] Goss PW, Paterson MA, Renalson J. A 'radical' new rural model for pediatric diabetes care. Pediatric diabetes 2010; 11: 296-304

[60] O'Connell MA, Donath S, Cameron FJ. Poor adherence to integral daily tasks limits the efficacy of CSII in youth. Pediatric diabetes 2011; 12: 556-559

[61] Deltsidou A. Age at menarche and menstrual irregularities of adolescents with type 1 diabetes. Journal of pediatric and adolescent gynecology 2010; 23: 162-167

[62] Rohan JM, Delamater A, Pendley JS, Dolan L, Reeves G, Drotar D. Identification of self-management patterns in pediatric type 1 diabetes using cluster analysis. Pediatric diabetes 2011; 12: 611-618

[63] Miller AR, Nebesio TD, DiMeglio LA. Insulin dose changes in children attending a residential diabetes camp. Diabetic medicine: a journal of the British Diabetic Association 2011; 28: 480-486

[64] Pingul MM RS, Gopalakrishnan G, Plante W, Boney CM, Quintos JBQ. Pediatric diabetes outpatient center at Rhode Island hospital: The impact of changing initial diabetes education from inpatient to outpatient. Endocrine Reviews 2011; 32

[65] Buckingham B NK, Benassi K, Realsen J, Liljenquist D, Chase P. Effectiveness and safety study of the prototype 4th generation seven day continuous glucose monitoring system in youth with type 1 diabetes mellitus. Diabetologia 2011; 54 [Suppl1]: 352

[66] Spiegel G, Bortsov A, Bishop FK et al. Randomized nutrition education intervention to improve carbohydrate counting in adolescents with type 1 diabetes study: is more intensive education needed? Journal of the Academy of Nutrition and Dietetics 2012; 112: 1736-1746

[67] Bergenstal RM, Tamborlane WV, Ahmann A et al. Effectiveness of sensor-augmented insulin-pump therapy in type 1 diabetes. The New England journal of medicine 2010; 363: 311-320

[68] Redondo MJ, Rodriguez LM, Escalante M, Smith EO, Balasubramanyam A, Haymond MW. Types of pediatric diabetes mellitus defined by anti-islet autoimmunity and random C-peptide at diagnosis. Pediatric diabetes 2013; 14: 333-340

[69] Malalasekera V, Cameron F, Grixti E, Thomas MC. Potential reno-protective effects of a gluten-free diet in type 1 diabetes. Diabetologia 2009; 52: 798-800

[70] Berg CA, King PS, Butler JM, Pham P, Palmer D, Wiebe DJ. Parental involvement and adolescents' diabetes management: the mediating role of self-efficacy and externalizing and internalizing behaviors. Journal of pediatric psychology 2011; 36: 329-339

[71] Wu YP, Graves MM, Roberts MC, Mitchell AC. Is insulin pump therapy better than injection for adolescents with diabetes? Diabetes research and clinical practice 2010; 89: 121-125

[72] Wysocki T, Iannotti R, Weissberg-Benchell J et al. Diabetes problem solving by youths with type 1 diabetes and their caregivers: measurement, validation, and longitudinal associations with glycemic control. Journal of pediatric psychology 2008; 33: 875-884

[73] Mehta SN, Volkening LK, Anderson BJ et al. Dietary behaviors predict glycemic control in youth with type 1 diabetes. Diabetes care 2008; 31: 1318-1320

[74] Wilkinson J, McFann K, Chase HP. Factors affecting improved glycaemic control in youth using insulin pumps. Diabetic medicine: a journal of the British Diabetic Association 2010; 27: 1174-1177

[75] Cengiz E WJ, Miller K. Resetting the bar: Frequency of severe hypoglycemia (SH) and diabetic ketoacidosis (DKA) among children with type 1 diabetes (T1D) in the T1D exchange registry. Clinical and Translational Science 2012; 12 [Suppl. 15]: 40-143

[76] Gerard-Gonzalez A, Gitelman SE, Cheng P et al. Comparison of autoantibody-positive and autoantibody-negative pediatric participants enrolled in the T1D Exchange clinic registry. Journal of diabetes 2013; 5: 216-223

[77] Criego A BK, Miller KM. Increased Body Mass Index (BMI) is associated with higher hemoglobin A1c (A1c) among 6-12 year olds but is not associated with total daily insulin dose per kg (TDI) in type 1 diabetes (T1D) participants enrolled in T1D Exchange Clinic Registry. Pediatric diabetes 2011; 12:34

[78] Lawrence JM AA, Imperatore G, Mayer-Davis EJ, Seid M, Waitzfelder B, Yi-Frazier J. Diabetes-related quality of life and glycaemic control among youth with type 1 diabetes. Diabetologia 2009; 52

[79] Gomez-Diaz RA, Ramirez-Soriano E, Tanus Hajj J et al. Association between carotid intima-media thickness, buccodental status, and glycemic control in pediatric type 1 diabetes. Pediatric diabetes 2012; 13: 552-558

[80] Markowitz JT, Laffel LM, Volkening LK et al. Validation of an abbreviated adherence measure for young people with Type 1 diabetes. Diabetic medicine: a journal of the British Diabetic Association 2011; 28: 1113-1117

[81] Rausch JR, Hood KK, Delamater A et al. Changes in treatment adherence and glycemic control during the transition to adolescence in type 1 diabetes. Diabetes care 2012; 35: 1219-1224

[82] Rovner AJ, Nansel TR, Mehta SN, Higgins LA, Haynie DL, Laffel LM. Development and validation of the type 1 diabetes nutrition knowledge survey. Diabetes care 2012; 35: 1643-1647

[83] Lukacs A, Kiss-Toth E, Varga B, Soos A, Takac P, Barkai L. Benefits of continuous subcutaneous insulin infusion on quality of life. International journal of technology assessment in health care 2013; 29: 48-52

[84] Markowitz JT, Butler DA, Volkening LK, Antisdel JE, Anderson BJ, Laffel LM. Brief screening tool for disordered eating in diabetes: internal consistency and external validity in a contemporary sample of pediatric patients with type 1 diabetes. Diabetes care 2010; 33: 495-500

[85] Lewin AB, LaGreca AM, Geffken GR et al. Validity and reliability of an adolescent and parent rating scale of type 1 diabetes adherence behaviors: the Self-Care Inventory (SCI). Journal of pediatric psychology 2009; 34: 999-1007

[86] Patton SR, Eder S, Schwab J, Sisson CM. Survey of insulin site rotation in youth with type 1 diabetes mellitus. Journal of pediatric health care: official publication of National Association of Pediatric Nurse Associates & Practitioners 2010; 24: 365-371

[87] Patton SR, Clements MA, Fridlington A, Cohoon C, Turpin AL, Delurgio SA. Frequency of mealtime insulin bolus as a proxy measure of adherence for children and youths with type 1 diabetes mellitus. Diabetes technology & therapeutics 2013; 15: 124-128

[88] Cortina S, Repaske DR, Hood KK. Sociodemographic and psychosocial factors associated with continuous subcutaneous insulin infusion in adolescents with type 1 diabetes. Pediatric diabetes 2010; 11: 337-344

[89] Hassan K, Heptulla RA. Glycemic control in pediatric type 1 diabetes: role of caregiver literacy. Pediatrics 2010; 125: e1104-1108

[90] Wiebe DJ, Gelfand D, Butler JM et al. Longitudinal associations of maternal depressive symptoms, maternal involvement, and diabetes management across adolescence. Journal of pediatric psychology 2011; 36: 837-846

[91] Herzer M, Hood KK. Anxiety symptoms in adolescents with type 1 diabetes: association with blood glucose monitoring and glycemic control. Journal of pediatric psychology 2010; 35: 415-425

[92] Ingerski LM, Laffel L, Drotar D, Repaske D, Hood KK. Correlates of glycemic control and quality of life outcomes in adolescents with type 1 diabetes. Pediatric diabetes 2010; 11: 563-571

[93] Wintergerst KA, Hinkle KM, Barnes CN, Omoruyi AO, Foster MB. The impact of health insurance coverage on pediatric diabetes management. Diabetes research and clinical practice 2010; 90: 40-44

[94] Graziano PA, Geffken GR, Williams LB et al. Gender differences in the relationship between parental report of self-regulation skills and adolescents' management of type 1 diabetes. Pediatric diabetes 2011; 12: 410-418

[95] Guilfoyle SM, Crimmins NA, Hood KK. Blood glucose monitoring and glycemic control in adolescents with type 1 diabetes: meter downloads versus self-report. Pediatric diabetes 2011; 12: 560-566

[96] Winsett RP, Stender SR, Gower G, Burghen GA. Adolescent self-efficacy and resilience in participants attending A diabetes camp. Pediatric nursing 2010; 36: 293-296

[97] Savoldelli R YM, Fontan F, Della Manna T, Menezes-Filho HC, Steinmetz L, Damiani D. Vitamin D insufficiency in a Brazilian type 1 diabetes mellitus pediatric population. . Pediatric diabetes 2010; 11 [Suppl. 14]: 90-91

[98] Calliari LE BM, Schechtman HP, Ribeiro EFA et al. Ten year evolution on diagnosis and treatment of type1 diabetes mellitus in an university center in Sao Paulo, Brazil. Pediatric diabetes 2009; 10: 58

[99] Tran F, Vu DC, Nguyen HT et al. Glycaemic control in children with neonatal diabetes and type 1 diabetes in Vietnam. International health 2011; 3: 188-192

[100] Al-Hussaini AA, Alenizi AS, AlZahrani MD, Sulaiman NM, Khan M. Is there an association between type 1 diabetes in children and gallbladder stones formation? Saudi journal of gastroenterology: official journal of the Saudi Gastroenterology Association 2013; 19: 86-88

[101] Mukama LJ, Moran A, Nyindo M, Philemon R, Msuya L. Improved glycemic control and acute complications among children with type 1 diabetes mellitus in Moshi, Tanzania. Pediatric diabetes 2013; 14: 211-216

[102] Juvenile Diabetes Research Foundation Continuous Glucose Monitoring Study Group. Effectiveness of continuous glucose monitoring in a clinical care environment: evidence from the Juvenile Diabetes Research Foundation continuous glucose monitoring (JDRF-CGM) trial. Diabetes care 2010; 33: 17-22

[103] Phillip M, Battelino T, Atlas E et al. Nocturnal glucose control with an artificial pancreas at a diabetes camp. The New England journal of medicine 2013; 368: 824-833

[104] de Wit M, Winterdijk P, Aanstoot HJ et al. Assessing diabetes-related quality of life of youth with type 1 diabetes in routine clinical care: the MIND Youth Questionnaire (MY-Q). Pediatric diabetes 2012; 13: 638-646

[105] Adolfsson P, Veijola R, Huot C, Hansen HD, Lademann JB, Phillip M. Safety and patient perception of an insulin pen with simple memory function for children and adolescents with type 1 diabetes--the REMIND study. Current medical research and opinion 2012; 28: 1455-1463
